# Towards DFO*^12^—Preliminary Results of a New Chelator for the Complexation of Actinium-225

**DOI:** 10.3390/pharmaceutics17030320

**Published:** 2025-03-01

**Authors:** Irene V. J. Feiner, Dennis Svatunek, Martin Pressler, Tori Demuth, Xabier Guarrochena, Johannes H. Sterba, Susanne Dorudi, Clemens Pichler, Christoph Denk, Thomas L. Mindt

**Affiliations:** 1 Bioinorganic Radiochemistry, Institute of Inorganic Chemistry, Faculty of Chemistry, University of Vienna, Währinger Straße 42, 1090 Vienna, Austriaxabier.guarrochena@univie.ac.at (X.G.); 2 Ludwig Boltzmann Institute Applied Diagnostics, AKH Wien c/o Sekretariat Nuklearmedizin, Währinger Gürtel 18-20, 1090 Vienna, Austria; 3 Division of Nuclear Medicine, Department of Biomedical Imaging and Image Guided Therapy, Medical University of Vienna, Währinger Gürtel 18-20, 1090 Vienna, Austria; 4 Institute of Applied Synthetic Chemistry, TU Wien, Getreidemarkt 9, 1060 Vienna, Austria; dennis.svatunek@tuwien.ac.at (D.S.); martin.pressler@tuwien.ac.at (M.P.); tori.demuth@tuwien.ac.at (T.D.); christoph.denk@tuwien.ac.at (C.D.); 5 Center for Labelling and Isotope Production, TRIGA Center Atominstitut, TU Wien, Stadionallee 2, 1020 Vienna, Austria; johannes.sterba@tuwien.ac.at; 6 Institute of Atomic and Subatomic Physics, TU Wien, Stadionallee 2, 1020 Vienna, Austria; 7 Vienna Doctoral School in Chemistry, University of Vienna, Währinger Straße 42, 1090 Vienna, Austria; 8 DSD Pharma GmbH, Schuhmeierstrasse 24, 1140 Purkersdorf bei Wien, Austriaclemens.pichler@dsd-pharma.com (C.P.); 9 Joint Applied Medicinal Radiochemistry Facility, University of Vienna and Medical University of Vienna, 1090 Vienna, Austria

**Keywords:** DFO*^12^, hydroxamate-based chelators, Actinium-225, solid-phase synthesis, DFT calculations, ^225^Ac-generator

## Abstract

**Background**: Actinium-225 (^225^Ac) has gained interest in nuclear medicine for use in targeted alpha therapy (TAT) for the treatment of cancer. However, the number of suitable chelators for the stable complexation of ^225^Ac^3+^ is limited. The promising physical properties of ^225^Ac result in an increased demand for the radioisotope that is not matched by its current supply. To expand the possibilities for the development of ^225^Ac-based TAT therapeutics, a new hydroxamate-based chelator, DFO*^12^, is described. We report the DFT-guided design of dodecadentate DFO*^12^ and an efficient and convenient automated solid-phase synthesis for its preparation. To address the limited availability of ^225^Ac, a small-scale ^229^Th/^225^Ac generator was constructed in-house to provide [^225^Ac]AcCl_3_ for research. **Methods**: DFT calculations were performed in ORCA 5.0.1 using the BP86 functional with empirical dispersion correction D3 and Becke–Johnson damping (D3BJ). The monomer synthesis over three steps enabled the solid-phase synthesis of DFO*^12^. The small-scale ^229^Th/^225^Ac generator was realized by extracting ^229^Th from aged ^233^U material. Radiolabeling of DFO*^12^ with ^225^Ac was performed in 1 M TRIS pH 8.5 or 1.5 M NaOAc pH 4.5 for 30 min at 37 °C. **Results**: DFT calculations directed the design of a dodecadentate chelator. The automated synthesis of the chelator DFO*^12^ and the development of a small-scale ^229^Th/^225^Ac generator allowed for the radiolabeling of DFO*^12^ with ^225^Ac quantitatively at 37 °C within 30 min. The complex [^225^Ac]Ac-DFO*^12^ indicated good stability in different media for 20 h. **Conclusions**: The novel hydroxamate-based dodecadentate chelator DFO*^12^, together with the developed ^229^Th/^225^Ac generator, provide new opportunities for ^225^Ac research for future radiopharmaceutical development and applications in TAT.

## 1. Introduction

Targeted alpha therapy (TAT) is a promising, emerging therapy regime in nuclear medicine for the specific and effective treatment of cancer [1,2,3,4,5,6]. The high linear energy transfer (LET) of α-particles results in an increased therapeutic efficacy compared to the currently more commonly used β^−^-radiotherapies (e.g., with Luthetium-177 (^177^Lu) and Yttrium-90 (^90^Y)) [7]. In this context, the clinical applications of α-particle emitters such as Astatine-211 (^211^At), Lead-212 (^212^Pb, in vivo generator of Bismuth-212 (^212^Bi)), Radium-223 (^223^Ra), Actinium-225 (^225^Ac), and Thorium-227 (^227^Th) have gained significant interest in nuclear medicine in recent years. ^223^RaCl_2_ (Xofigo^®^), for the treatment of osteoplastic bone metastases originating from castration-resistant prostate cancer [8], was the first alpha particle-emitting drug approved by the FDA and EMA in 2013. In the beginning of 2024, ^212^Pb-DOTAMTATE (AlphaMedix^TM^) was granted a breakthrough therapy designation for the treatment of adults with advanced gastroenteropancreatic neuroendocrine tumors (GEP-NETs) [9]. In particular, the application of ^225^Ac has shown very promising results in clinical trials for the treatment of cancer [2,10]. The decay of ^225^Ac leads to a chain reaction in which several α- and β-particles are emitted until stable Bismuth-209 is reached. This enables the delivery of a high dose to the tumor tissue for efficient therapy, and due to the short range of alpha particles (50–100 µm) [11], non-targeted tissue can be spared. The lack of a stable actinium isotope does not facilitate experimental research concerning its complexation chemistry; however, steady progress has been made in this field ([12], xy). Other important aspects to be addressed for the medical applications of ^225^Ac include the consideration of dosimetry [2,13], the potential release of daughter isotopes from the radiopharmaceutical caused by the recoil energy resulting from an alpha decay [2], as well as the appropriate analytical methods for monitoring the radiolabeling reactions of an alpha particle emitter and its decay chain. In addition, the limited supply of ^225^Ac cannot meet its pre-clinical and clinical demand [5]. To circumvent this limitation, we constructed a small-scale ^229^Th/^225^Ac generator in-house to provide [^225^Ac]AcCl_3_ reliably in relatively small amounts, but amounts sufficient for preliminary radiochemical research. Due to these constraints and the inherent challenges with the detectability of ^225^Ac, stability experiments could only be performed in this study for 20 h and not, as often performed for planned applications with antibodies, up to seven or more days.

For applications in TAT, it is inevitable to have chelators available that form thermodynamically and kinetically stable complexes in vivo [14] for transporting the radionuclide specifically to tumors and metastases using biological vectors such as tumor-targeting peptides or antibodies. To date, only a small number of chelators for ^225^Ac have been reported in the literature. In aqueous solution, ^225^Ac is found in the oxidation state of +3 and it is the largest +3 ion found in the periodic table. According to Pearson’s HSAB theory, ^225^Ac^3+^ belongs to the category of hard Lewis acids [15]. Hence, it predictably prefers chelators with hard, electronegative donor atoms such as oxygen. Today, the most commonly used chelator for ^225^Ac^3+^ is DOTA (dodecane tetraacetic acid; 2,2′,2″,2‴-(1,4,7,10-tetraazacyclododecane-1,4,7,10-tetrayl)tetraacetic acid). However, the ionic radius of ^225^Ac^3+^ (the six-coordinate ionic radius of Ac^3+^ is 1.12 Å) [15] does not fit well with the small macrocyclic cage of DOTA. It has been shown that the thermodynamic stability of the complexes of a metal ion with DOTA depends on the ionic radius, with less stable complexes given with larger metal ions [15]. Furthermore, the high temperature required for the formation of [^225^Ac]Ac-DOTA is a disadvantage when working with temperature-sensitive biomolecules such as antibodies. A number of structurally diverse chelators for ^225^Ac^3+^ have been examined and reported in recent years; however, the majority lack either radiolabeling efficiency or the stability of the corresponding ^225^Ac-complex under physiological conditions [15,16,17,18,19,20,21]. It is noteworthy that Thiele and co-workers developed MacroPa [17], an eighteen-membered macrocyclic ligand for ^225^Ac, which is able to form a complex with ^225^Ac^3+^ at room temperature in 5 min. The bifunctional MacroPa-NCS was successfully conjugated to target biomolecules and showed high stability in vivo in mice. Preclinical studies were performed in LNCaP xenograft mice, using [^225^Ac]Ac-macropa-RPS-070 [17] and in HepG2 liver cancer tumor-bearing mice using [^225^Ac]Ac-GC33-macropa and [^225^Ac]Ac-GC33-BZmacropa [18]. Next to DOTA and MacroPa, it would be advantageous to have more candidates available for studying the effect of different coordination modes of the chelator, as well as the charge, size, and hydrophilicity of different ^225^Ac complexes. In addition, a larger variety of synthetically readily available chelators could facilitate the functionalization, adaptability for imaging companion, and translatability. 

We report herein the development of a novel, acyclic, dodecadentate ^225^Ac-chelator DFO*^12^ composed of multiple oxygen-containing hydroxamate groups that are connected by a linking aliphatic chain of appropriate length. Hydroxamate moieties are well-studied bidentate chelating units suitable for incorporation into multidentate ligand systems [22] for the complexation of radiometals; however, they have not yet been examined in the context of ^225^Ac^3+^. The design of DFO*^12^ was supported by DFT calculation, offering insights into the denticity and bonding characteristics of the chelator [23,24]. The preparation of DFO*^12^ was accomplished by an efficient and convenient automated solid-phase synthesis approach enabled by the application of an appropriate protective group strategy. The new chelator DFO*^12^ is shown to exhibit promising properties for the future development of radiopharmaceuticals adequate for applications in TAT.

## 2. Materials and Methods

### 2.1. Materials

#### 2.1.1. DFT Calculations

DFT calculations were performed in ORCA 5.0.1 [25] using the BP86 functional [26,27] with empirical dispersion correction D3 and Becke–Johnson damping (D3BJ) [28]. This density functional was found to provide good results for actinide species while being computationally efficient [29]. The relativistic effects were incorporated using the Zero-Order Regular Approximation (ZORA) [30]. The solvent effects were treated implicitly using the Conductor-like Polarizable Continuum Model (CPCM) [31] with water as the solvent. The calculations were accelerated using the RI-J method as implemented in ORCA. The ZORA-def2-SVP [32] basis set was used for all atoms except actinium, for which the SARC-ZORA-TZVP [33] basis set was employed. The SARC/J [33,34] auxiliary basis set was used for density fitting.

#### 2.1.2. Monomer and Chelator Synthesis

Reagents were purchased from Sigma-Aldrich (Buchs, Switzerland), Merck (Darmstadt, Germany), abcr (Karlsruhe, Germany), Acros Organics (Geel, Belgium), Fluorochem (Hadfield, UK), Novabiochem (Darmstadt, Germany), TCI (Zwijndrecht, Belgium), and Fluka (Buchs, Switzerland), and were used without further purification. MacroPa was obtained by the synthesis described in the literature [17]. [^225^Ac]AcCl_3_ was used from the generator described in this work. In the case of increased demand, [^225^Ac]AcCl_3_ was ordered through DSD Pharma, Austria. For the human serum stability assays, pooled human serum off the clot was used from Innovative^TM^ Research. The TLC plates used for monitoring the reactions of the monomer synthesis were purchased from Macherey-Nagel, Düren, Germany (DC-Fertigfolien ALUGRAM^®^ Xtra SIL G/UV_254_). The chelators were synthesized using solid-phase Fmoc (Fluorenylmethoxycarbonyl) chemistry on the microwave peptide synthesizer Biotage^®^ Initiator + Alstra™. RP-HPLC-MS analyses were conducted at 220 nm on an Agilent 1260 Infinity II system (Agilent Technologies, Vienna, Austria) equipped with a Flexible pump, a 1260 VWD UV-Vis detector, and the LC-MSD system using the Acquity UPLC^®^ BEH C18 column (300 Å, 1.7 μm, 2.1 mm × 100 mm). Mobile phase A was H_2_O (0.1% formic acid (FA)) and mobile phase B was acetonitrile (ACN) (0.1% FA). The flowrate was 0.6 mL/min. For preparative RP-HPLC, a Waters XBridge C18 column was used on an Agilent 1200 Series system (Agilent Technologies, Vienna, Austria). The methods used are described in the respective protocols. The HRESI-MS spectra for the monomer synthesis (compound **2**–**4**) (*m*/*z* 50–1900) were obtained on a maXis UHR ESI-Qq-TOF mass spectrometer (Bruker Daltonics, Bremen, Germany) in the positive-ion mode by direct infusion. The HRESI-MS spectra for the chelator (compound **5**) (*m*/*z* 20–40,000) were obtained on an ESI/MALDI-Qq-TOF with a dual-TIMS analyzer mass spectrometer (Bruker Daltonics, Bremen, Germany) in the positive-ion mode by direct infusion. The sum formulae of the detected ions were determined using Bruker Compass DataAnalysis 4.1 based on the mass accuracy (Δ*m*/*z* ≤ 5 ppm) and isotopic pattern matching (SmartFormula algorithm). Nuclear magnetic resonance (NMR) measurements were recorded either on a Bruker FT-NMR Avance III 500 MHz or 600 MHz spectrometer at 298 K in deuterated methanol (MeOH-d4), deuterated chloroform (CDCl_3_), or deuterated dimethyl sulfoxide (DMSO-d6). The respective residual solvent peak was used as an internal reference for the chemical shifts (ppm). The spin multiplicities are abbreviated as follows: s = singlet, d = doublet, t = triplet, q = quartet, m = multiplet, and bs = broad signal. The values of the coupling constants (*J*) are given in Hertz (Hz). For centrifugation, a ROTINA 380/380R from Hettich (Tuttlingen, Germany) was used. Microwave reactions were performed with a Biotage Initiator+ system. The device has a range of power up to 400 W and needs about 1 min to heat to the desired temperature.

#### 2.1.3. ^229^Th/^225^Ac Generator

For the ^225^Ac generator, nitric and hydrochloric acid (ROTIPURAN^®^Supra) were purchased from Carl Roth^®^, Karlsruhe, Germany, and were used without further purification. Triple distilled water was prepared in-house. The strong anionic resins Dowex^®^ 1 × 8 100–200 mesh (Carl Roth^®^) and AG MP-1M 100–200 mesh (Bio-Rad, Hercules, CA, USA) were washed chloride free using dilute nitric acid before preconditioning. DGA and UTEVA resin (Triskem, Bruz, France) and Sep-Pak^®^ C18 Cartridges (Waters Corporation, Milford, MA, USA) were used without further purification. Alpha spectrometry was performed with a partially depleted PIPS detector from Canberra Industries (22 keV FWHM for α, 600 mm^2^ active area, and 100 μm thickness). Samples were prepared by the evaporation of a small volume of liquid on a stainless steel disc (Triskem). The evaluation was performed by comparing the samples to known ^232^Th and ^233^U reference materials using Maestro Multi-Channel Analyzer software (Version 6.08 for Windows) from ORTEC^®^ (Oak Ridge, TN, USA). Gamma spectrometry was performed with a 151 cm^3^ HPGe-detector from Canberra Industries (Meriden, CT, USA, 1.8 keV resolution at the 1332 keV ^60^Co peak; 50.1% relative efficiency), connected to a PC-based multi-channel analyzer with a preloaded filter and Loss-Free Counting (LFC) system. The Genie 2000 Basic Spectroscopy Software version 3.4 from Canberra (RRID:SCR_021933) was used for evaluation.

#### 2.1.4. Radiochemistry

To determine the amount of radioactivity used in each reaction, a dose calibrator (iDose, Elysia Raytest, Angleur, Belgium) was utilized. For the analysis of the radiolabeling reactions, iTLC-SG plates (instant Thin-layer chromatography paper impregnated with a silica gel, Agilent Technologies, Vienna, Austria) were used. The Radio-TLC-reader was a miniGITA Dual from Elysia Raytest (Angleur, Belgium), set up with a gamma detector (OFA Probe).

### 2.2. Methods

#### 2.2.1. Monomer Synthesis

**Amino aldehyde 2**. To a 1 M solution of DMSO (2.2 eq., 30.5 mmol, 2.4 mL) in anhydrous dichloromethane (30 mL), a 2 M solution of oxalyl chloride (1.2 eq., 18.5 mmol, 1.6 mL) in anhydrous dichloromethane (8.5 mL) was added dropwise over 5 min at −45 °C (dry ice/acetonitrile). The reaction was stirred for 10 min and a 0.65 M solution of the Fmoc-amino pentanol **1** (1.0 eq., 15.0 mmol, 5.0 g) in anhydrous dichloromethane (23.0 mL) was slowly added at −45 °C. The reaction was stirred for another 30 min at −45 °C, followed by the addition of N,N-diisopropylethylamine (DIPEA, 3 eq., 45.0 mmol, 8.0 mL), after which the temperature was allowed to increase to −20 °C and the mixture was stirred for 90 min. The reaction was monitored by thin-layer chromatography (TLC, hexane/ethyl acetate 7:3, R_f_ (alcohol **1**) = 0.06, R_f_ (aldehyde **2**) = 0.28). Upon completion of the reaction, as indicated by TLC, the solvent was removed in vacuum, and the crude product was dissolved in dichloromethane and washed three times each with 1 M KHSO_4_ and brine. The organic phase was dried over Na_2_SO_4_, filtered, and the solvent was removed under reduced pressure to yield the amino aldehyde **2** as yellow oil. Crude amino aldehyde **2** was used directly for the next step without purification.

**Hydroxylamine 3**. A 0.1 M solution of the amino aldehyde **2** (1.0 eq., 15.0 mmol) was prepared in methanol (50 mL). *O*-*t*Bu-hydroxylamine-hydrochloride (3.0 eq., 45.0 mmol, 5.6 g) was added and the mixture stirred at room temperature overnight. The completion of the reaction was verified by TLC (hexane/ethyl acetate 8:2, R_f_ (product) = 0.26) and the solvent was evaporated under reduced pressure (for the characterization of the hydroxylimine intermediate **3_IM_**, see Supporting Information (SI), Figures S2–S4). The crude product was dissolved in methanol and the reduction was conducted by adding NaBH_3_CN (4.0 eq., 60.0 mmol, 3.8 g) and acetic acid (5.0 eq., 75.0 mmol, 4.3 mL) at room temperature. The reaction was stirred for 3.5 h at room temperature and the conversion to the desired hydroxylamine was confirmed by TLC (hexane/ethyl acetate 8:2, R_f_ (product) = 0.17). After the evaporation of methanol, the crude product was dissolved in dichloromethane and washed three times each with sat. NaHCO_3_ and brine. The organic phase was dried over Na_2_SO_4_, filtered, and the solvent was removed under reduced pressure to yield the hydroxylamine **3** as yellow oil. The crude product was analyzed by NMR and MS (for the characterization of the hydroxylamine, see SI, Figures S5–S7). Hydroxylamine **3** was used directly for the next step without purification.

^1^*H NMR* (600 MHz, CDCl_3_) δ 7.78–7.74 (m, 2H), 7.61–7.58 (m, 2H), 7.42–7.37 (m, 2H), 7.33–7.29 (m, 2H), 4.41 (d, J = 6.8 Hz, 2H), 4.22 (t, J = 6.6 Hz, 1H), 3.23–3.15 (m, 2H), 2.85 (t, J = 7.0 Hz, 2H), 1.55–1.46 (m, 4H), 1.40–1.33 (m, 2H), and 1.17 (s, 9H) ppm. The NH proton was not observed, likely due to overlapping signals.

^13^*C-NMR* (151 MHz, CDCl_3_, DEPTq135) δ 156.42, 144.04, 141.34, 127.66, 127.03, 125.04, 119.98, 76.68, 66.48, 52.83, 47.34, 41.00, 29.93, 27.03, 26.83, and 24.49 ppm.

*HRESI-MS m*/*z* found (calculated) for C_24_H_32_N_2_O_3_ is as follows: [M + H]^+^ 397.2491 (397.2486).

**Fmoc-mon(*t*Bu) 4**. The hydroxylamine **3** (1.0 eq., 15.0 mmol) was dissolved in dimethylformamide (DMF, 10 mL). Succinic anhydride (5.0 eq., 75.0 mmol, 7.6 g) and 4-(dimethylamino)pyridin (DMAP, 0.5 eq., 7.5 mmol, 0.9 g) were added at room temperature. The reaction was stirred for 90 min at 50 °C and the conversion to the desired Fmoc-mon(*t*Bu) was monitored by TLC (hexane/ethyl acetate 1:1 + 1% acetic acid, R_f_ (product) = 0.32) and HPLC-MS (5–95% ACN/H_2_O over 6 min with 0.1% FA). DMF was removed under high vacuum at 40 °C and the crude product was dissolved in methanol for its subsequent purification via preparative HPLC (flow rate: 17 mL/min, mobile phase A = H_2_O + 0.1% FA, mobile phase B = MeOH + 0.1% FA, gradient: 0–1.5 min 75% B, 10.0 min 85% B, 10.5 min 95% B, 14.5 min 95% B, 15.0 min 75% B). The fractions containing the product were pooled and lyophilized to yield Fmoc-mon(*t*Bu) **4** as a white powder with an overall yield over three steps of 33.5% (for the full characterization of the monomer **4**, see SI, Figures S8–S14).

^1^*H NMR* (600 MHz, CDCl_3_) δ 7.79–7.72 (m, 2H), 7.63–7.55 (m, 2H), 7.44–7.35 (m, 2H), 7.34–7.28 (m, 2H), 6.42 (s, 1H), 5.07–4.96 (m, 1H), 4.39 (d, J = 7.0 Hz, 2H), 4.32–4.10 (m, 1H), 3.23–3.14 (m, 2H), 2.84–2.72 (m, 1H), 2.68–2.58 (m, 1H), 1.72–1.58 (m, 1H), 1.58–1.37 (m, 2H), 1.32 (s, 9H), and 1.29–1.16 (m, 3H) ppm. Some CH_2_ signals were not observed due to overlapping signals (see SI for more information).

^13^*C-NMR* (151 MHz, CDCl_3_, DEPTq135) δ 177.18, 175.97, 156.76, 144.15, 141.45, 127.78, 127.18, 125.21, 120.09, 83.18, 67.59, 66.73, 47.42, 41.66, 41.04, 29.64, 29.30, 27.96, 23.72, and 23.56 ppm.

*HRESI-MS m*/*z* found (calculated) for C_28_H_36_N_2_O_6_ is as follows: [M + Na]^+^ 519.2470 (519.2466).

#### 2.2.2. Chelator Synthesis

DFO*^12^ 5. The synthesis of the chelator DFO*^12^ **5** was performed automatically with the microwave-assisted peptide synthesizer Biotage^®^ Initiator + Alstra™ by standard Fluorenylmethoxycarbonyl (Fmoc) chemistry. The reaction was carried out in a 10 mL vial at a scale of 0.03–0.05 mmol. The low loading Rink amide MBHA (4-methylbenzylhydrylamine) resin (0.041 mmol/g loading, 100–200 mesh) was used as the solid support. The resin was swollen in DMF at 70 °C for 20 min. The cleavage of the Fmoc protecting group was accomplished using 2 × 4.5 mL 20% piperidine in DMF at room temperature for a 3- and 10-min reaction time, respectively. After Fmoc-deprotection, the resin was washed with DMF (2 × 4.5 mL). The monomer (2 eq.) was prepared in 0.04 M solutions in DMF. HATU (hexafluorophosphate azabenzotriazole tetramethyl uronium, 0.05 M in DMF, 2 eq.) and DIPEA (0.05 M in DMF, 5 eq.) were used as coupling reagents. The coupling reactions were carried out for 20 min at 60 °C. After the first coupling of Fmoc-mon(*t*Bu) **4** to the resin, a capping step was carried out as follows: Acetic anhydride (5 M in DMF, 180 eq.) was added to the reaction vial for 10 min at room temperature. The coupling cycles (Fmoc deprotection, followed by coupling) were repeated five times. The last coupling was conducted as double coupling. After the completion of the sequence, the final Fmoc was removed, and the resin was washed with DCM (3 × 3 mL). The cleavage from the resin and the deprotection of the chelator were performed using a microwave reactor in TFA/H_2_O/TIPS (38:1:1, *v*:*v*:*v*) for 1 h at 45 °C. The chelator was dried under a stream of argon, precipitated with ice-cold diethyl ether (Et_2_O, 1 mL), centrifuged (5 °C, 5000 rpm, 5 min), and decanted. After one additional wash with ice-cold Et_2_O followed by sonication, centrifugation, and decanting, the crude product was isolated and analyzed by HPLC-MS (10–45% ACN/H_2_O over 6 min with 0.1% FA, crude yield of approx. 55%) and purified by preparative HPLC (flow rate: 17 mL/min, mobile phase A = H_2_O + 0.1% FA, mobile phase B = CAN + 0.1% FA, gradient: 0–5 min 10% B, 20.0 min 40% B, 20.5 min 95% B, 24.0 min 95% B, and 24.5 min 10% B). After lyophilization, the product **5** was isolated as a white solid with an overall yield of approx. 4% (approx. 2 mg) and a purity, according to HPLC, of >95% (for the analytical data of the chelator **5**, see SI, Figures S15–S19).

^1^*H NMR* (500 MHz, DMSO) δ 9.67–9.47 (m, 5H), 7.86–7.64 (m, 5H), 7.55 (s, 2H), 7.24 (s, 1H), 6.68 (s, 1H), 3.46–3.38 (m, 13H), 2.99–2.91 (m, 9H), 2.74–2.69 (m, 1H), 2.61–2.58 (m, 2H), 2.56–2.49 (m, 9H), 2.33–2.30 (m, 3H), 2.26–2.18 (m, 11H), 1.51–1.40 (m, 13H), 1.37–1.27 (m, 10H), and 1.22–1.12 (m, 13H) ppm. One expected NH signal was not observed, likely due to overlapping signals.

*HRESI-MS m*/*z* found (calculated) for C_54_H_99_N_13_O_18_ is as follows: [M + H]^+^ 1218.7285 (1218.7304).

#### 2.2.3. ^229^Th/^225^Ac Generator

**Isolation of ^229^Th from ^233^U and construction of a ^229^Th/^225^Ac generator.**

A glass column with frit was dry loaded with strong anion-exchange resin (20 g, Dowex^®^ 1X8 100–200 mesh) and equilibrated with HNO_3_ (8 M). A solution of ^233^U (160 mg) dissolved in HNO_3_ (32 mL, 8 M) was passed through the equilibrated column. The column was then washed extensively with HNO_3_ (8 M) to remove the remaining unbound uranium.

The built-up radionuclides were eluted with HNO_3_ (26 mL, 8 M) and the resulting eluate was loaded onto AG MP-1M resin (1.0 g, 100–200 mesh, Bio-Rad), preconditioned with HNO_3_ (8 M). The eluate was evaporated to dryness and redissolved in HNO_3_ (4 mL, 4 M). A stack of two cartridges were prepared, with the top cartridge containing UTEVA resin (0.5 g, 100–150 µm, Triskem) and the bottom one with normal DGA resin (0.75 g, 100–150 µm, Triskem). After preconditioning with HNO_3_ (4 M), the stack was loaded and washed with HNO_3_ (7 mL, 4 M), resulting in a radium fraction, which was stored for later use. The cartridges were separated and the DGA cartridge washed with HCl (7.5 mL, 8 M) to remove the lead isotopes. Before the elution of ^225^Ac, a Sep-Pak^®^ C18 column preconditioned with methanol and water was installed on the end of the DGA cartridge to remove organic impurities. ^225^Ac was recovered from the DGA resin using HCl (7.5 mL, 2 M). The eluate was evaporated to dryness and reconstituted in HCl (0.2 mL, 0.1 M). After a prolonged time (>2 years), a moderate breakthrough was observed (3% per elution). This fact was mitigated by the aforementioned ion exchange step (AG MP-1M resin), which allowed for the recycling of eluted ^229^Th.

#### 2.2.4. Radiochemistry

Radiosynthesis of [^225^Ac]Ac-DFO*^12^ and [^225^Ac]Ac-Macropa

A stock solution of DFO*^12^ (0.01 mg/µL, 8.2 mM) was prepared in DMSO for later use. [^225^Ac]AcCl_3_ (~60 kBq) was transferred to a 0.5 mL reaction vial and diluted with buffer (1 M TRIS buffer pH 8.5 or 1.5 M NaOAc buffer pH 4.5) to a total volume of approx. 90 µL. A total of 10 μL of the chelator stock solution was added to the solution of ^225^Ac, resulting in a final volume of 100 µL (final concentration of DFO*^12^ = 0.8 mM, pH was stable at 8.5 or 4.5, respectively). The mixture was incubated at 37 °C for 30 min in a heating block. The progress of the reaction was monitored by radioTLC (iTLC-SG; see SI, Figures S20–S22) with 50 mM EDTA*2Na in water (pH 4.5) as the mobile phase. The radiolabeling of Macropa with [^225^Ac]AcCl_3_ was performed in analogy.

Stability assay of [^225^Ac]Ac-DFO*^12^ in PBS

The ^225^Ac-complex of DFO*^12^ was prepared with a chelator concentration of 0.8 mM in buffer (1 M TRIS-buffer pH 8.5 or 1.5 M NaOAc-buffer pH 4.5) as described above (30 min at 37 °C). A total of 50 μL (40 nmol) of the solution were transferred to a LoBind Eppendorf^®^ tube containing 450 μL of PBS (pH 7.4). The sample was gently shaken at 37 °C. RadioTLCs (iTLC-SG, 50 mM EDTA*2Na, pH 4.5) of the mixture were performed at different time points up to 20 h to determine the stability of the [^225^Ac]Ac-DFO*^12^ complex (see SI, Figures S23 and S24).

Stability assay of [^225^Ac]Ac-DFO*^12^ in human blood serum

The ^225^Ac-complex of DFO*^12^ was prepared with a chelator concentration of 0.8 mM in buffer (1 M TRIS buffer pH 8.5 or 1.5 M NaOAc buffer pH 4.5) as described above (30 min at 37 °C). A total of 50 μL (40 nmol) of the solution was transferred to a LoBind Eppendorf^®^ tube containing 450 μL blood serum (pooled human serum off the clot, Innovative Research). The sample was gently shaken at 37 °C. RadioTLCs (iTLC-SG, 50 mM EDTA*2Na, pH 4.5) of the mixture were performed at different time points up to 20 h to determine the stability of the [^225^Ac]Ac-DFO*^12^ complex (see SI, Figure S25).

## 3. Results and Discussion

### 3.1. DFT Calculations

**Figure 1:**
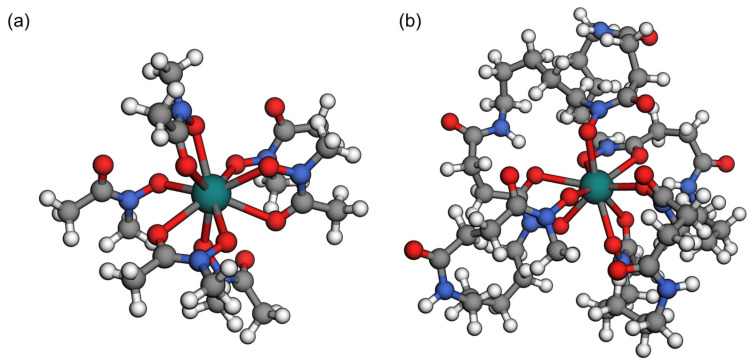
BP86-D3 (BJ)/ZORA-def2-SVP-SARC-ZORA-TZVP (Ac) CPCM (water)-calculated structures of (**a**) a model complex with unconnected aceto-N-methylhydroxamates and (**b**) a DFO*^12^-Ac^3+^ complex in which the hydroxamate moieties are connected through a linker of nine atoms including C, O, and N. Gray = carbon, blue = nitrogen, red = oxygen, white = hydrogen, and teal = actinium.

DFT calculations were performed to gain an insight into the bonding characteristics of the new chelator DFO*^12^ with ^225^Ac^3+^. Due to the countless possible diastereomers and structural isomers, exploring the full conformational and configurational space was not feasible. Therefore, we focused on gaining qualitative insights into the bonding and structural properties of the chelator, rather than quantitative ones. To investigate bonding with an up to dodecadentate ligand, we used six individual hydroxamate units, specifically aceto-N-methylhydroxamate ligands (for structure see SI, Figure S1a) with a central Ac^3+^ ion. The extensive study of this model complex revealed that the maximum coordination number reached was nine (Figure 1a), with eight also occurring (for an example, see SI, Figure S1b). In both cases, all of the hydroxamate ligands coordinated with the actinium ion via the hydroxylamine oxygen (distance 2.65 ± 0.05 Å), while either two or three of the carbonyl oxygens also coordinated (distance approx. 2.75 ± 0.05 Å). This result might suggest that a decadendate ligand would be sufficient; however, the hydroxylamine O-^225^Ac coordination was stronger than the carbonyl coordination. Therefore, using six hydroxamate units coordinating the metal in a mono- or bidentate fashion could still provide a benefit.

Next, starting from the complex shown in Figure 1a, we incorporated an aliphatic linking chain consisting of nine atoms, including an amide functionality between the individual hydroxamate units to construct the full DFO*^12^ chelator. Both the cis and trans configurations of the amide backbones were explored. The resulting complex (Figure 1b) showed only marginal differences in bonding to the actinium ion, maintaining a coordination number of nine. This confirms that a linker length of nine atoms provides sufficient flexibility of the chelator for the optimal spatial arrangement of the hydroxamate units around the metal center.

### 3.2. Monomer and Chelator Synthesis

**Scheme 1:**
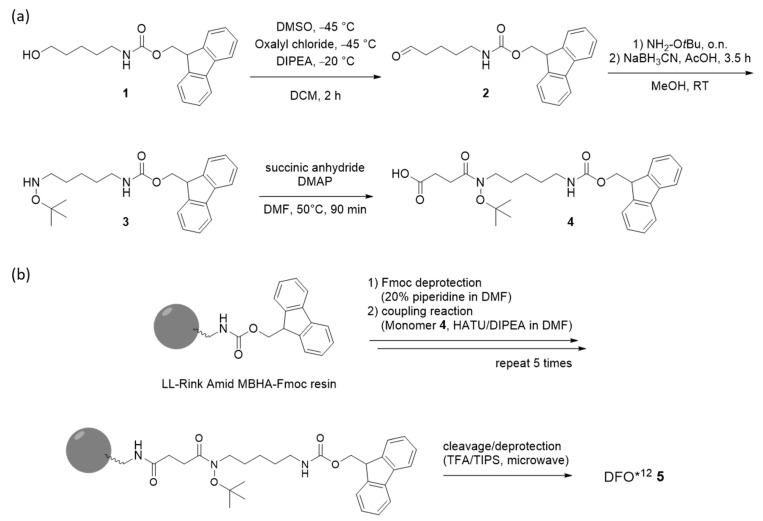
(**a**) Synthesis of the monomer Fmoc-mon(tBu) **4**. (**b**) Solid-phase synthesis of the chelator DFO*^12^ **5**.

**Figure 2:**
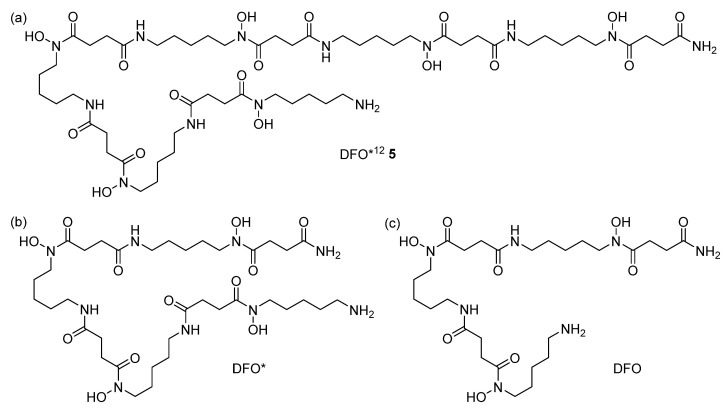
Structure of the new dodecadentate DFO*^12^ **5** (**a**) and the related hydroxamate chelators octadentate DFO* (**b**) and hexadentate DFO (**c**) [22].

Building on our previous experiences with the synthesis of poly-hydroxamate-containing chelators [35], we aimed to prepare a dodecadentate chelator that combines the benefits of a solid-phase synthesis approach reported for oxoDFO* [36,37] with an appropriate protective group strategy (*t*Bu instead of Bn), as recently published by us for the improved fragmentation condensation synthesis of DFO* (Figure 2b) [38]. The application of the former would allow for the straightforward, potentially large-scale automated synthesis of the chelator, whereas the latter would avoid the formation of dehydroxylated side-products, as observed in the case of the use of Bn-protective groups. In addition, the use of *t*Bu-protective groups enables the simultaneous deprotection of the hydroxamate groups and the cleavage of the chelator from the resin in a single step. The synthesis of a suitable monomer (Fmoc-mon(*t*Bu) **4**, Scheme 1a) started with the Swern oxidation [39] of commercial Fmoc-protected amino pentanol **1**. The obtained aldehyde **2** was subsequently subjected to a reductive amination [40] using a *t*Bu-hydroxylamine and sodium cyanoborohydride. Acylation of the hydroxylamine **3** by a reaction with succinic anhydride provided the desired monomer Fmoc-mon(*t*Bu) **4** in three steps, whereas the purification of intermediates was not required. The final purification by preparative HPLC afforded compound **4** with an overall yield starting from the alcohol **1** of 33.5% and a purity of >95%, according to HPLC.

With Fmoc-mon(*t*Bu) **4** obtained, the automated solid-phase synthesis of DFO*^12^ **5** was adapted to and optimized for the use of a Biotage^®^ Initiator + Alstra™ peptide synthesizer (Scheme 1b). Using a low loading Rink Amide MBHA resin, various coupling conditions were tested, including the use of different coupling agents, as well as reaction temperatures and times (data shown in SI, Table S1). The best results were obtained with HATU and DIPEA within 20 min at 60 °C using microwave heating. After six coupling cycles, the deprotection and simultaneous cleavage of DFO*^12^ **5** from the resin was investigated. The reaction time was shown to be crucial to ensure the complete removal of protective groups and the avoidance of the formation of side products. The optimized conditions were found using a cleavage cocktail of TFA/H_2_O/TIPS (38:1:1, *v*:*v*:*v*) and microwave heating at 45 °C for 60 min. The crude product (yield of approx. 55% according to HPLC) was purified via preparative HPLC. The challenging HPLC purification induced a significant loss of the product and provided DFO*^12^ (Figure 2a) in only low yields (approx. 4%) after lyophilization. To improve the yield of the isolated DFO*^12^ by, e.g., 5–10-fold, the efficiency of the purification of the final product DFO*^12^ still needed to be improved, e.g., by applying a different purification strategy such as the use of Sep-Pak cartridges, as we have reported recently for the purification of DFO* [38]. DFO*^12^ **5** was obtained as a white powder with an excellent purity of >95% (SI, Figure S15).

### 3.3. ^229^Th/^225^Ac Generator

To enable radiolabeling experiments with the new chelator DFO*^12^, despite the limited availability of the alpha particle emitter ^225^Ac, a small-scale ^229^Th/^225^Ac generator was successfully realized in-house by extracting ^229^Th from aged ^233^U material. The procedures were adapted from previous work reported by Morgenstern and co-workers. [41] and the Eichrom Technical Note [42]. The generator could be harvested for 120 kBq ^225^Ac every 30 days or for 220 kBq ^225^Ac every 70 days. The workup of the collected radium fraction after 13–24 days yielded, additionally, up to 90 kBq or 42% of ^225^Ac activity relative to the primary fraction.

According to the α-spectroscopy measurement, the uranium solution also contained ^232^U with an activity ratio of 25:1 between ^233^U and ^232^U. The uranium material, in the form of uranyl nitrate (UO_2_(NO_3_)_2_), dissolved in concentrated nitric acid and was stored for decades, resulting in a build-up of ^229^Th and ^228^Th from ^233^U and ^232^U, respectively. The presence of ^232^U had no influence on the isotopic purity of the final product since there were no actinium isotopes present in its decay chain.

**Figure 3:**
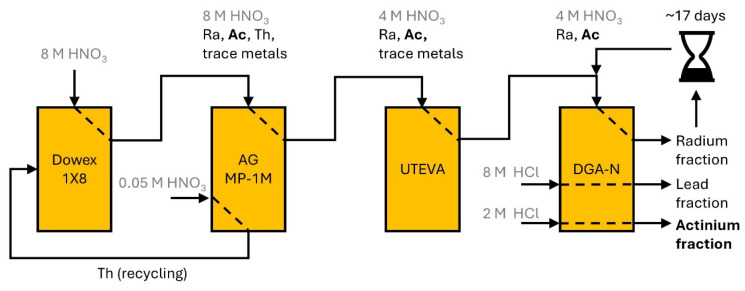
Separation scheme of ^225^Ac from ^229^Th generator.

To separate ^225^Ac from the decay products of both decay series, multiple ion exchange and extraction chromatography steps were undertaken, which are summarized in Figure 3. Thorium was separated from uranium using a strong anion-exchange resin (Dowex^®^ 1X8 100–200 mesh). At high nitric acid concentrations (8 M), thorium is strongly retained, while uranium only shows minimal retention. The decay products of ^229^Th, namely ^225^Ra and ^225^Ac, were allowed to build up on the generator for several weeks.

The elution of the decay products was performed using nitric acid (8 M) and the eluate was directly loaded onto an anion-exchange resin (AG MP-1M) to remove any breakthrough of thorium. An extraction resin (UTEVA) was used to separate uranium, thorium, and trace metals, before loading the elute directly onto a final extraction resin (DGA). There, radium was directly eluted and actinium and other decay products were retained. After the removal of unwanted decay products (mainly lead), actinium was readily eluted in diluted hydrochloric acid (2 M).

**Figure 4:**
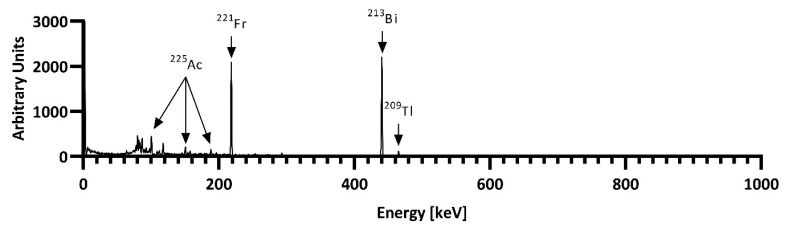
Representative γ-spectra of an ^225^Ac solution after reaching equilibrium, with its daughters ^221^Fr and ^213^Bi showing a radionuclidic purity of >98%.

After 13–24 days, the last extraction chromatography step was repeated with the radium fraction to extract the built-up ^225^Ac. All fractions were analyzed for their radioisotopic purity using γ-spectroscopy. ^225^Ac was quantified via its decay product ^221^Fr (218.0 keV) after a sufficient buildup time (>35 min, Figure 4). Since it is impractical to detect ^225^Ra itself, the emissions of ^224^Ra (241.0 keV) and its decay products ^212^Pb (238.6 keV) and ^212^Bi (727.3 keV) were used to investigate the successful separation of radium, and γ-spectroscopy showed a radionuclide purity of >98%.

### 3.4. Radiochemistry

With the hydroxamate-based dodecadentate chelator DFO*^12^ and ^225^Ac^3+^ being available, we moved on to investigate and optimize the radiolabeling conditions. The first task was to identify a suitable radioTLC system for ^225^Ac and/or its daughter nuclides. Due to the combination of the challenge of detecting α-particles (low range) and the decay chain of ^225^Ac with several α-, β-, and γ-emissions leading to several radioactive daughter nuclides, the analysis of the radiolabeling processes is complex. It is anticipated that the daughter nuclides do not form a complex with the chelator, due to their unique coordination chemistry [20]. Yet, a distribution of those across the TLC plate is likely, due to the loosely formed complexes of the daughter nuclides with the chelator and/or the mobile phase. It is recommended to wait for the secular equilibrium between Actinium-225 and its daughter radionuclides before performing quality control tests. However, the commonly referenced time point of up to 20 h is impractical, and thus, shorter times have been evaluated [43]. In 2024, the IAEA published new guidelines for the ‘Production and Quality Control of Actinium-225 Radiopharmaceuticals’, confirming that a 30 min time point after developing the radioTLC is sufficient to reach the secular equilibrium to Francium-221 (six half-lives of the daughter to be measured), and therefore to evaluate the radioTLC [44].

For the experiments described in this work, we found reliable and reproducible results by using silica gel-impregnated radioTLC sheets (iTLC-SG, Agilent) in combination with an aqueous mobile phase of 50 mM EDTA. A clear separation of the product [^225^Ac]Ac-DFO*^12^ (R_f_ = 0, baseline) and free ^225^Ac^3+^ in the form of [^225^Ac]Ac-EDTA (R_f_ = 1, solvent front) was achieved. We performed the readout of tlc sheets by a radio tlc-reader at different time points after development, ranging from 0 min to approx. 3 h. Even though the IAEA guideline recommends the analysis of ^225^Ac-based radiopharmaceuticals by tlc after 30 min, we did not observe significant differences.

**Figure 5:**
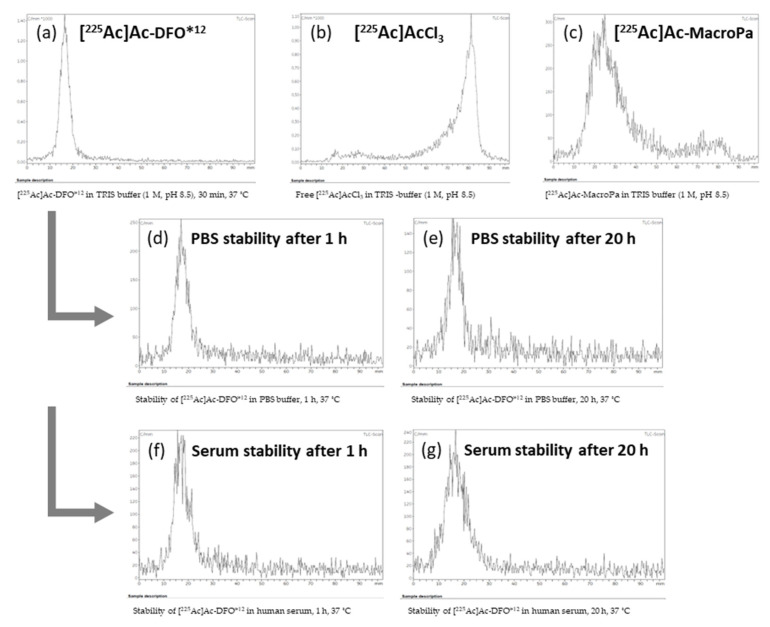
First row: RadioTLC chromatograms (iTLC-SG, eluent: 50 mM EDTA) of radiolabeling experiments. Signal at R_f_ = 0 represents ^225^Ac-chelator complexes. Signal at R_f_ = 1 represents [^225^Ac]Ac-EDTA. (**a**) Labeling solution of [^225^Ac]Ac-DFO*^12^ in TRIS buffer (1 M, pH 8.5) after 30 min at 37 °C. (**b**) Free [^225^Ac]AcCl_3_ in TRIS buffer (1 M, pH 8.5). (**c**) [^225^Ac]Ac-MacroPa in TRIS buffer (1 M, pH 8.5). For radioTLC chromatograms of NaOAc buffer, see SI (Figures S20–S22). Second row: Stability assay in PBS buffer of [^225^Ac]Ac-DFO^*12^, radiolabeled in TRIS buffer (1 M, pH 8.5) at 37 °C. (**d**) After 1 h. (**e**) After 20 h. For radioTLC chromatograms of NaOAc buffer (1.5 M, pH 4.5), see SI (Figure S24). Third row: Stability assay in human serum of [^225^Ac]Ac-DFO*^12^, radiolabeled in TRIS buffer (1 M, pH 8.5) at 37 °C. (**f**) After 1 h. (**g**) After 20 h. For radioTLC chromatograms of NaOAc buffer (1.5 M, pH 4.5), see SI (Figure S25).

To identify the optimum radiolabeling conditions, a variety of parameters such as different buffers (ammonium acetate, HEPES, TRIS, sodium acetate), temperatures (room temperature to 95 °C), pH (4.5 to 8.5), and reaction times (30 min to 24 h) were investigated (Table 1). The best results at neutral pH (7.4) were achieved in TRIS buffer (1 M) at 60 °C with a 12% radiochemical yield (RCY) after 3.5 h. However, no improvements could be made by elongating the reaction time. The best conditions to radiolabel DFO*^12^ with [^225^Ac]AcCl_3_ were found in TRIS (1 M, pH 8.5) or sodium acetate (1.5 M, NaOAc, pH 4.5) buffer. Quantitative labeling could be achieved in these buffers after 30 min at 37 °C (n = 2; Figure 5a) at a chelator concentration of 0.8 mM.

As a control, two reference compounds were investigated and analyzed by the TLC system used: (1) free [^225^Ac]AcCl_3_ in the respective buffers (Figure 5b; expected R_f_ = 1 as in the EDTA complex) and (2) [^225^Ac]Ac-MacroPa [17] (Figure 5c; expected R_f_ = 0 as in the case of [^225^Ac]Ac-DFO*^12^) radiolabeled under the same conditions. A comparison of the results confirmed the successful radiolabeling of the new chelator DFO*^12^ with ^225^Ac ([^225^Ac]Ac-DFO*^12^; R_f_ = 0, Figure 5a) under mild reaction conditions in quantitative RCY after 30 min. The stability of the [^225^Ac]Ac-DFO*^12^ was determined in PBS buffer and human blood serum for the products obtained from both buffers investigated (TRIS and NaOAc; n = 2–3). All experiments indicated the good stability of the complex (>95%) over 20 h (Figure 5d,e and SI, Figures S23–S25). A determination of the stabilities at time points later than 20 h was not performed, because we envision the application of DFO*^12^ in combination with small molecules (e.g., PSMA ligands) and peptides, which exhibit fast pharmacokinetics (usually renal clearance within a few hours).

We have shown that DFO*^12^ is an interesting new chelator for ^225^Ac providing [^225^Ac]Ac-DFO*^12^ quantitatively under mild reaction conditions in two different buffer systems, and which is stable in different media. In the next step, we investigate the bifunctionalization of DFO*^12^, its conjugation to biological vectors (e.g., tumor-targeting peptides), and further studies of the resulting ^225^Ac-DFO*^12^ conjugates, including their stability at later points and their preclinical biological evaluation. This continuation of our work, for which funding is pending, will be reported in due time.

## 4. Conclusions

In summary, we report the novel hydroxamate-based, dodecadentante, acyclic chelator DFO*^12^, the design of which was guided by DFT calculations. The convenient solid-phase synthesis and high ^225^Ac-radiolabeling efficiency of DFO*^12^, together with the observed stability of [^225^Ac]Ac-DFO*^12^ in different media, makes it a promising candidate for further radiopharmaceutical development applicable to TAT. We also demonstrate that a small-scale, in-house-constructed ^225^Ac-generator can provide the means for radiochemical/pharmaceutical research independent from the commercial sources of the still scarcely available alpha emitter ^225^Ac.

## Figures and Tables

**Table 1:** Exemplary results of experiments to determine the optimum radiolabeling conditions for DFO*^12^ with [^225^Ac]AcCl_3_. The complete conditions and results of radiolabeling reactions can be found in the SI (Table S2, page S21). All experiments were performed with a final volume of 100 µL, an activity of 60 kBq (1.6 µCi), and 0.1 mg chelator (0.8 mM). NaOAc (sodium acetate), NH_4_OAc (ammonium acetate), HEPES (4-(2-hydroxyethyl)-1-piperazineethanesulfonic acid), and TRIS (tris(hydroxymethyl)aminomethane). Quant. = quantitative. RCY = radiochemical yield. The color code in the column of pH refers to the pH range; red is acidic, green is physiological and blue is basic.

Buffer	pH	Temperature (°C)	Time (h)	RCY
NaOAc (1.5 M)	4.5	37	0.5	quant.
NH_4_OAc (1 M)	7.3	60	24.0	-
HEPES (0.5 M)	7.4	60	24.0	-
TRIS (1 M)	7.4	60	3.5	12%
TRIS (1 M)	8.5	37	0.5	quant.

## Data Availability

All data generated or analyzed during this study are included in this published article and its Supplementary Information Files.

## Supplementary Materials

The following supporting information can be downloaded at: www.mdpi.com/article/10.3390/pharmaceutics17030320/s1, Figure S1: DFT calculation–structures and model compounds; Figure S2: ^1^H-NMR spectrum of hydroxylimine **3_IM_**; Figure S3: ^13^C-NMR spectrum of hydroxylimine **3_IM_**; Figure S4: HRMS spectrum of hydroxylimine **3_IM_**; Figure S5: ^1^H-NMR spectrum of hydroxylamine **3**; Figure S6: ^13^C-NMR spectrum of hydroxylamine **3**; Figure S7: HRMS spectrum of hydroxylamine **3**; Figure S8: ^1^H-NMR spectrum of Fmoc-mon(tBu) **4**; Figure S9: ^13^C-NMR spectrum of Fmoc-mon(tBu) **4**; Figure S10: COSY-NMR spectrum of Fmoc-mon(tBu) **4**; Figure S11: HSQC-NMR spectrum of Fmoc-mon(tBu) **4**; Figure S12: NOESY-NMR spectrum of Fmoc-mon(tBu) **4**; Figure S13: HMBC-NMR spectrum of Fmoc-mon(tBu) **4**; Figure S14: HRMS spectrum of Fmoc-mon(tBu) **4**; Table S1: Tested coupling reaction conditions; Figure S15: LC chromatogram of a) crude DFO*^12^ **5** after cleavage and b) purified DFO*^12^ **5**; Figure S16: HRMS spectrum of DFO*^12^ **5**; Figure S17: ^1^H-NMR spectrum of DFO*^12^ **5**; Figure S18: COSY-NMR spectrum of DFO*^12^ **5**; Figure S19: NOESY-NMR spectrum of DFO*^12^ **5**; Table S2: Conditions and results of radiolabeling reactions; Figure S20: Radio-TLC of labelling solutions of **[^225^Ac]Ac-DFO^*12^**; Figure S21: Radio-TLC of free **[^225^Ac]AcCl_3_**; Figure S22: Radio-TLC of labelling solutions of **[^225^Ac]Ac-MacroPa**; Figures S23 and S24: Radio-TLC of stability assay in PBS; Figure S25: Radio-TLC of stability assay in human serum.

## Abbreviations

Bn	Benzyl
DCM	Dichloromethane
DFO	Desferrioxamine B
DFT	Density functional theory
DIPEA	N,N-diisopropylethylamine
DMF	Dimethylformamide
DMSO	Dimethyl sulfoxide
DOTA	Dodecane tetraacetic acid
EDTA	Ethylenediaminetetraacetic acid
FA	Formic acid
Fmoc	Fluorenylmethoxycarbonyl
HATU	Hexafluorophosphate azabenzotriazole tetramethyl uronium
HRESI-MS	High-resolution electrospray mass spectrometry
MBHA	Methylbenzhydryl amine
NMR	Nuclear magnetic resonance spectroscopy
RCY	Radiochemical yield
Rf	Retention factor
RP-HPLC	Reversed-phase high-performance liquid chromatography
SI	Supporting information
SM	Starting material
TAT	Targeted alpha therapy
*t*Bu	*tert*-Butyl
TLC	Thin-layer chromatography
