# Supplementary material for: Towards DFO*12—Preliminary Results of a New Chelator for the Complexation of Actinium-225
Source: Pharmaceutics. 2025 Mar 1;17(3):320. doi: 10.3390/pharmaceutics17030320 (PMC11946154; doi:10.3390/pharmaceutics17030320)
Supplement: Supplementary file 1 [file pharmaceutics-17-00320-s001.zip › pharmaceutics-3397973-supplementary.pdf]

Supporting Information for

DFT-Guided Design and Efficient Solid Phase Syntheses of DFO*[12], a New Chelator for the Stable Complexation of Actinium-225

Irene V. J. Feiner, Dennis Svatunek, Martin Pressler, Tori Demuth, Xabier Guarrochena, Johannes H. Sterba, Clemens Pichler, Susanne Dorudi, Christoph Denk, Thomas L. Mindt

Table of content

Part 1: DFT calculations S1

1.1 Structures of model compounds S1

2.1 3D structures of model complex and full complex S2

Part 2: Monomer synthesis - NMR and HRMS Spectra

2.1 Hydroxylimine $\mathbf{3_{IM}}$ S9

2.2 Hydroxylamine **3** S11

2.3 Fmoc-mon(*t*Bu) **4** S13

Part 3: Chelator synthesis - LCMS/HRMS/NMR Spectra of the chelator **DFO*[12] 5** S17

Part 4: RadioTLC chromatograms of [^{225}Ac]Ac-DFO*[12]

4.1 Radiolabeling S21

4.2 Stability S22

Part 1: DFT calculations

1.1 **Structures of model compounds**

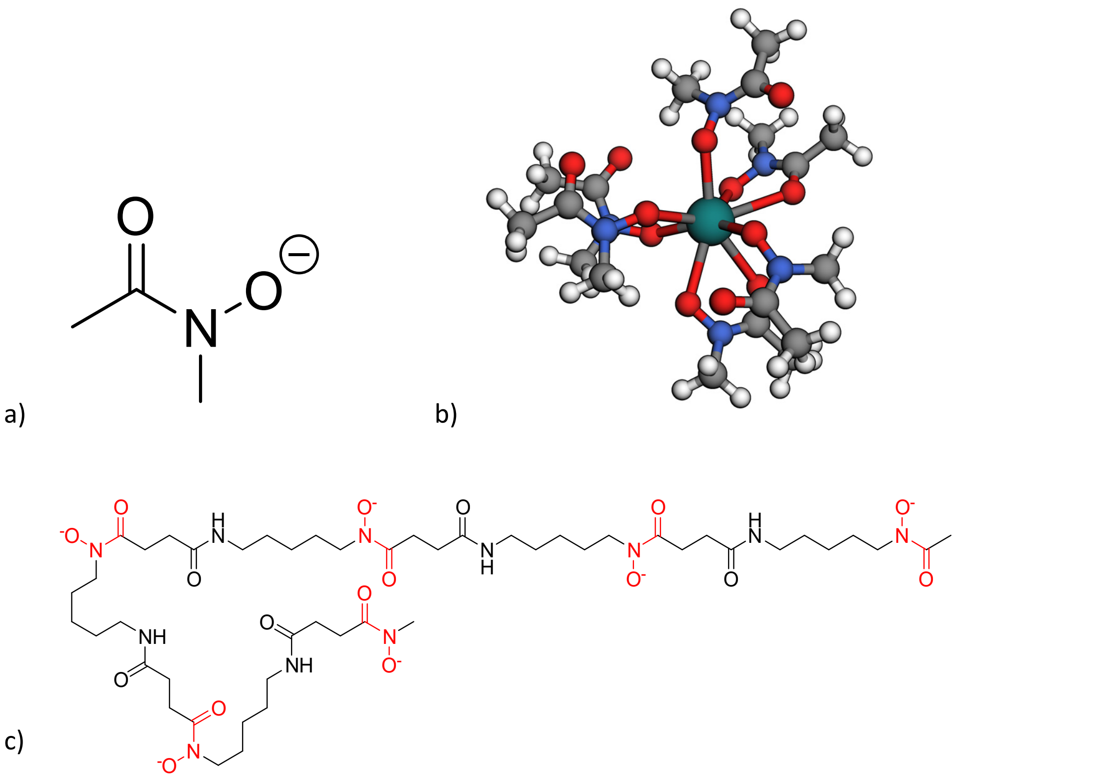

Figure S1: DFT calculation – structures and model compounds. a) Chemical structure of the aceto-N-methylhydroxamate anion; b) BP86-D3(BJ)/ZORA-def2-SVP-SARC-ZORA-TZVP(Ac) CPCM(water) calculated structure of a model complex with a coordination number of 8. grey = carbon, blue = nitrogen, red = oxygen, white = hydrogen, teal = actinium; c) Chemical structure of the DFO[12] anion, used to investigate linker geometries.*

1.2 **3D structures of model complex and full complex**

For the investigation of binding modes, the Ac^{3+} hexakis(aceto-N-methylhydroxamate) complex was used, which is denoted as **model complex** below. For the investigation of linkers between the individual hydroxamate units the DFO*[12] ligand shown in Figure S1c was used, this ligand is denoted as **full complex**.

Model complex

Ac	0.038144000	0.339049000	-0.055024000
O	1.291710000	0.967421000	2.098442000
O	0.254822000	0.471864000	4.572308000
O	-4.436193000	1.506944000	0.967390000
N	1.918984000	0.196845000	3.018456000
C	1.360294000	-0.026613000	4.232732000
C	2.124992000	-0.934357000	5.202197000

H	3.165856000	-0.608273000	5.379243000
H	1.582198000	-0.911681000	6.160353000
C	3.127648000	-0.436539000	2.518824000
H	3.624650000	-1.041729000	3.291191000
H	2.866684000	-1.086589000	1.663735000
O	-1.956584000	0.478002000	1.581387000
N	-3.080326000	-0.281097000	1.490782000
C	-4.284006000	0.281441000	1.216790000
C	-2.907140000	-1.673351000	1.898203000
C	-5.484914000	-0.668673000	1.204452000
H	-3.601349000	-2.340698000	1.363103000
H	-1.866711000	-1.955674000	1.657805000
H	-3.065938000	-1.800966000	2.990400000
H	-5.626375000	-1.193578000	2.167419000
H	-6.380493000	-0.060534000	1.001084000
O	-0.425061000	2.884870000	-0.059364000
N	0.258711000	3.825152000	-0.762405000
C	1.187946000	4.601926000	-0.151839000
C	-0.146197000	3.922393000	-2.156037000
O	1.482353000	4.479034000	1.065948000
C	1.894529000	5.652736000	-1.015318000
H	0.520727000	4.581348000	-2.731104000
H	-0.116543000	2.903898000	-2.587071000
H	2.469040000	5.198916000	-1.843599000
H	2.597407000	6.189075000	-0.358518000
O	0.301551000	-1.825813000	1.466427000
N	1.099524000	-2.837911000	1.086299000
C	1.877594000	-2.705375000	-0.005518000
C	1.070647000	-3.987442000	1.975732000
O	1.842089000	-1.623652000	-0.679426000
C	2.795538000	-3.846784000	-0.414509000
H	1.740723000	-4.787914000	1.629493000
H	1.378039000	-3.668889000	2.990498000
H	3.514018000	-4.115359000	0.381305000
H	2.234373000	-4.760481000	-0.684719000
H	3.363294000	-3.514354000	-1.297555000
O	0.700690000	0.783856000	-2.595611000
N	1.852886000	1.410165000	-2.926635000
C	2.721637000	1.763996000	-1.959303000
C	1.996730000	1.686158000	-4.345261000
O	2.480495000	1.469810000	-0.748526000
C	3.994777000	2.506193000	-2.339398000

H	2.971528000	2.141656000	-4.574353000
H	1.900411000	0.739367000	-4.909483000
H	3.784172000	3.464378000	-2.848254000
H	4.648937000	1.909590000	-3.001582000
O	-1.216006000	-1.668309000	-1.230292000
C	-3.207770000	-2.795376000	-1.751829000
H	-4.253265000	-2.658183000	-2.061830000
H	-2.683617000	-3.409214000	-2.509335000
O	-2.238878000	0.710264000	-1.567777000
C	-4.441867000	-0.038492000	-2.162738000
H	-4.451710000	0.487595000	-3.135891000
H	-5.077175000	-0.932328000	-2.246082000
N	-2.511089000	-1.530896000	-1.596115000
C	-3.004894000	-0.285805000	-1.753531000
H	1.195430000	6.386782000	-1.456917000
H	-1.183913000	4.309335000	-2.230556000
H	4.543130000	2.719940000	-1.408888000
H	2.156605000	-1.982700000	4.848239000
H	3.834282000	0.339398000	2.164514000
H	0.038087000	-4.380147000	2.034342000
H	-5.398008000	-1.440356000	0.417313000
H	-4.880659000	0.644618000	-1.413033000
H	-3.186613000	-3.345335000	-0.791608000
H	1.192301000	2.369906000	-4.680189000

Full complex

Ac	0.064802000	0.376642000	-0.096841000
O	1.363991000	0.779068000	2.023883000
O	0.273108000	1.745849000	4.285929000
O	-4.397908000	1.281831000	1.182950000
N	1.705809000	0.292872000	3.235567000
C	1.140448000	0.833183000	4.345380000
C	1.580802000	0.346434000	5.728226000
H	2.410343000	-0.377992000	5.711629000
H	1.964428000	1.239954000	6.257875000
C	2.597937000	-0.869937000	3.150083000
H	2.839200000	-1.248626000	4.154271000
H	2.014266000	-1.644352000	2.615124000
O	-1.858535000	0.372638000	1.595472000
N	-2.950462000	-0.410375000	1.767637000
C	-4.194007000	0.105954000	1.592409000
C	-2.613852000	-1.716772000	2.328265000

C	-5.392289000	-0.809936000	1.844421000
H	-3.416421000	-2.454312000	2.186609000
H	-1.700654000	-2.061785000	1.811105000
H	-2.381426000	-1.633424000	3.408943000
H	-5.166111000	-1.661866000	2.506274000
H	-6.157007000	-0.197710000	2.357213000
O	-0.599224000	2.895172000	0.260859000
N	0.173082000	3.747053000	-0.469869000
C	1.266700000	4.346540000	0.076395000
C	-0.395063000	4.088921000	-1.768201000
O	1.591419000	4.229412000	1.285754000
C	2.196222000	5.076383000	-0.896170000
H	0.354837000	4.639056000	-2.355270000
H	-0.581245000	3.139534000	-2.306919000
H	2.279830000	4.472686000	-1.815528000
H	3.193067000	5.053644000	-0.426418000
O	0.431450000	-1.952679000	1.160547000
N	1.154204000	-2.912602000	0.556224000
C	1.902007000	-2.600235000	-0.519008000
C	1.235870000	-4.204923000	1.255269000
O	1.927157000	-1.401666000	-0.959094000
C	2.696823000	-3.695122000	-1.211458000
H	1.509658000	-4.978179000	0.520334000
H	2.062478000	-4.159166000	1.995903000
H	3.520739000	-4.083028000	-0.583810000
H	2.065127000	-4.554806000	-1.501142000
H	3.131299000	-3.262888000	-2.126647000
O	0.544766000	1.119223000	-2.631067000
N	1.759033000	1.573323000	-3.003977000
C	2.687459000	1.812881000	-2.060527000
C	1.965309000	1.648642000	-4.454207000
O	2.422467000	1.571312000	-0.836949000
C	4.050349000	2.387645000	-2.453014000
H	3.046435000	1.698402000	-4.669115000
H	1.590986000	0.687800000	-4.855146000
H	3.916584000	3.408349000	-2.858165000
H	4.504376000	1.798166000	-3.272203000
O	-1.067456000	-1.620020000	-1.392041000
C	-2.924869000	-2.844206000	-2.176066000
H	-3.955375000	-2.686000000	-2.530990000
H	-2.356675000	-3.292261000	-3.016556000
O	-2.248479000	0.687826000	-1.623410000

C	-4.323370000	-0.225856000	-2.536526000
H	-4.293758000	-0.349233000	-3.638634000
H	-4.948205000	-1.053400000	-2.161951000
N	-2.328284000	-1.550996000	-1.858343000
C	-2.911543000	-0.347638000	-1.973684000
C	-0.075742000	-4.591346000	1.953071000
H	-0.916465000	-4.314861000	1.290959000
H	-0.096058000	-5.695904000	2.041153000
C	-0.282610000	-3.972653000	3.345464000
H	-0.154706000	-2.880367000	3.258331000
H	-1.329513000	-4.147615000	3.662268000
C	0.651418000	-4.527048000	4.426984000
H	1.710656000	-4.326824000	4.167421000
H	0.548397000	-5.630002000	4.481258000
C	0.369060000	-3.963000000	5.828630000
H	-0.709629000	-4.048738000	6.055261000
H	0.920179000	-4.551707000	6.585309000
N	0.728540000	-2.559517000	6.010560000
H	1.695244000	-2.355745000	6.262563000
C	-0.178256000	-1.541701000	6.068056000
O	-1.385404000	-1.702939000	5.820363000
C	0.379573000	-0.193164000	6.529154000
H	0.655882000	-0.290362000	7.597275000
H	-0.446337000	0.533298000	6.457761000
C	3.864389000	-0.556120000	2.349846000
H	3.539811000	-0.004027000	1.453352000
H	4.527015000	0.124282000	2.922691000
C	4.621742000	-1.827561000	1.949462000
H	4.987708000	-2.339659000	2.862554000
H	3.905108000	-2.530266000	1.475944000
C	5.802353000	-1.609732000	0.989406000
H	6.479045000	-0.822435000	1.376888000
H	6.402990000	-2.539642000	0.947620000
C	5.418515000	-1.263232000	-0.463091000
H	4.660056000	-1.976386000	-0.826160000
H	6.317541000	-1.366991000	-1.097916000
N	4.894021000	0.077641000	-0.680826000
C	5.715624000	1.128967000	-0.945916000
O	6.956076000	1.023172000	-0.964945000
H	3.878334000	0.247754000	-0.639022000
C	5.020360000	2.454940000	-1.259991000
H	4.466145000	2.803011000	-0.368971000

H	5.825092000	3.177409000	-1.471624000
C	1.239757000	2.817939000	-5.134479000
H	1.216831000	2.620958000	-6.224101000
H	0.186918000	2.821864000	-4.791551000
C	1.885330000	4.180003000	-4.859656000
H	1.886544000	4.343642000	-3.766137000
H	2.950510000	4.157351000	-5.165441000
C	1.182580000	5.359441000	-5.543595000
H	1.307523000	5.292890000	-6.642142000
H	0.091773000	5.300000000	-5.351959000
C	1.694510000	6.734384000	-5.070295000
H	1.215598000	7.543345000	-5.651954000
H	2.786608000	6.815989000	-5.211928000
N	1.440533000	6.939060000	-3.648894000
H	0.465510000	6.971061000	-3.351726000
C	2.393124000	6.773994000	-2.682692000
O	3.606728000	6.703741000	-2.935106000
C	1.847513000	6.543471000	-1.278027000
H	2.322592000	7.245768000	-0.570315000
H	0.757790000	6.718801000	-1.239928000
C	-1.654499000	4.969503000	-1.638428000
H	-2.492125000	4.368000000	-1.239897000
H	-1.427999000	5.737707000	-0.873564000
C	-2.054557000	5.675093000	-2.954412000
H	-2.371634000	6.710604000	-2.727265000
H	-1.149292000	5.772836000	-3.587245000
C	-3.153862000	5.026473000	-3.807842000
H	-4.130323000	5.083012000	-3.290461000
H	-3.264430000	5.625923000	-4.732611000
C	-2.922870000	3.556021000	-4.211279000
H	-3.467027000	3.360641000	-5.154923000
H	-1.850855000	3.369943000	-4.405077000
N	-3.380626000	2.587503000	-3.225168000
H	-2.728136000	2.035090000	-2.631182000
C	-4.693845000	2.253352000	-3.132934000
O	-5.584682000	2.810355000	-3.804292000
C	-5.013729000	1.106032000	-2.172917000
H	-4.759032000	1.376396000	-1.128588000
H	-6.107094000	0.972350000	-2.225486000
C	-2.906716000	-3.790987000	-0.964301000
H	-1.883395000	-4.179507000	-0.825123000
H	-3.125920000	-3.191649000	-0.060651000

C	-3.910004000	-4.950176000	-1.098443000
H	-3.793909000	-5.422800000	-2.093584000
H	-3.660199000	-5.736951000	-0.359598000
C	-5.374989000	-4.519032000	-0.899908000
H	-5.583421000	-3.601900000	-1.485933000
H	-6.063843000	-5.292854000	-1.288281000
C	-5.711771000	-4.245626000	0.578394000
H	-5.757782000	-5.200244000	1.132739000
H	-4.902262000	-3.663144000	1.052146000
C	-5.980327000	-1.311357000	0.505150000
H	-6.320664000	-0.439854000	-0.078872000
H	-5.188940000	-1.814896000	-0.074554000
N	-6.977976000	-3.549617000	0.783926000
H	-7.819545000	-4.115155000	0.910708000
C	-7.197586000	-2.202501000	0.718475000
O	-8.332150000	-1.720222000	0.870719000

Part 2: NMR and HRMS Spectra

2.1 **Hydroxylimine 3_{IM}**

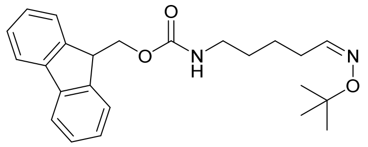

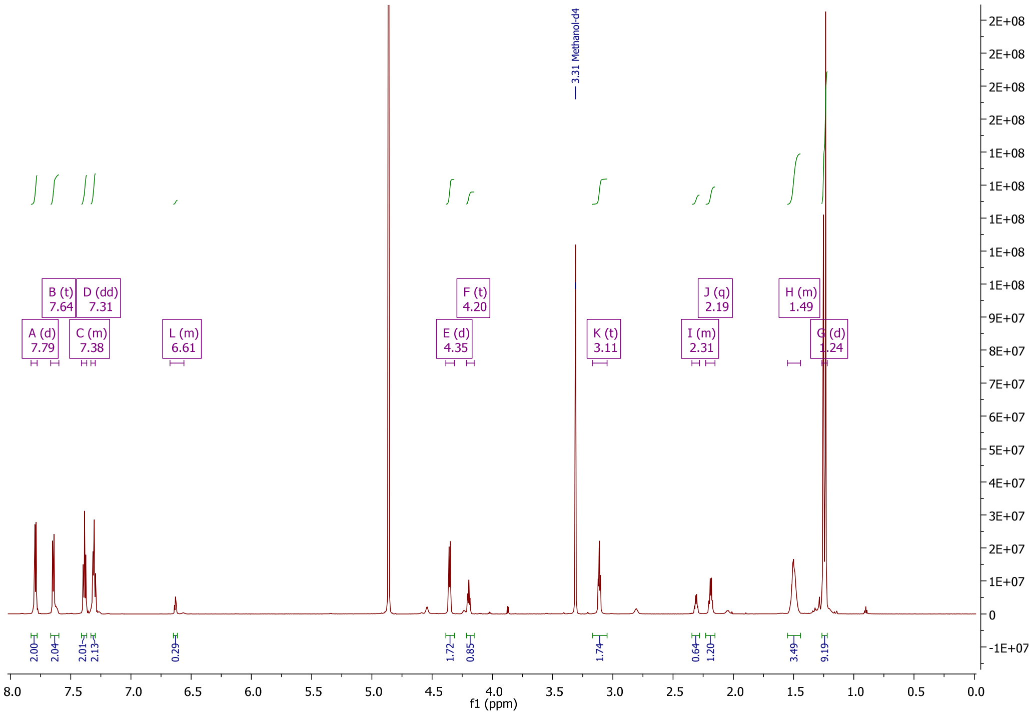

Figure S2: ^{1}H-NMR (700.40 MHz, MeOD) spectrum of hydroxylimine ***3_{IM}****. ^{1}H NMR (700 MHz, MeOD) δ 7.83 – 7.78 (m, 2H), 7.67 – 7.60 (m, 2H), 7.41 – 7.37 (m, 2H), 7.33 – 7.30 (m, 2H), 6.68 – 6.56 (m, 1H), 4.35 (d, J = 6.8 Hz, 2H), 4.20 (t, J = 6.8 Hz, 1H), 3.17 – 3.05 (m, 2H), 2.34 – 2.28 (m, 1H), 2.23 – 2.15 (m, 1H), 1.55 – 1.44 (m, 4H), 1.26-1.22 (m, 9H) ppm. The NH proton was not observed due to H/D exchange in MeOH-d_4.*

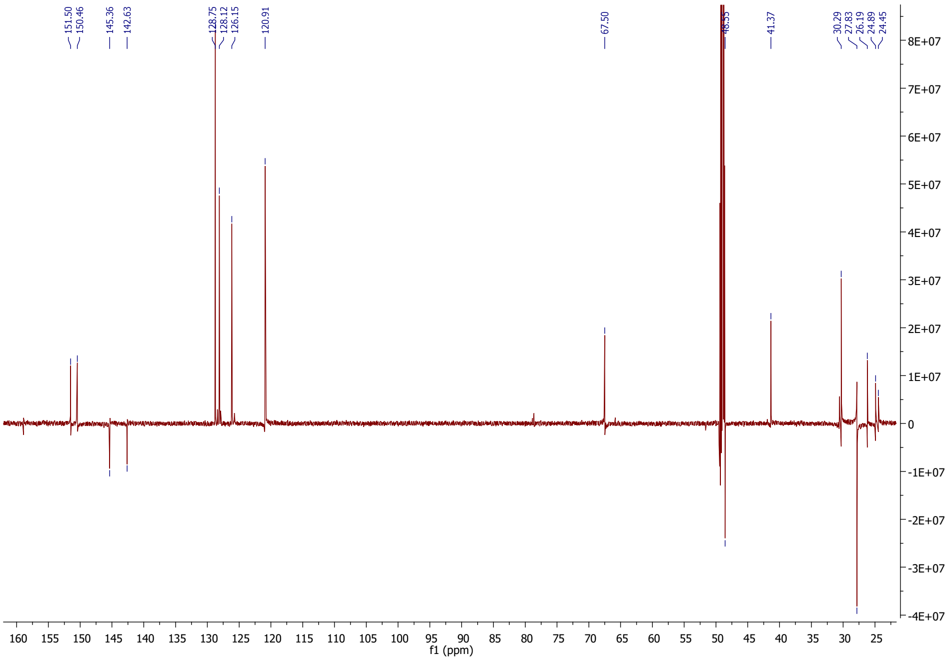

*Figure S3: ^{13}C-NMR (176 MHz, MeOD, DEPTq135) spectrum of hydroxylimine **3**$_{IM}$. ^{13}C-NMR (176 MHz, MeOD, DEPTq135) δ 151.50, 150.46, 145.36, 142.63, 128.75, 128.12, 126.15, 120.91, 67.50, 48.55, 41.37, 30.29, 27.83, 26.19, 24.89, 24.45 ppm.*

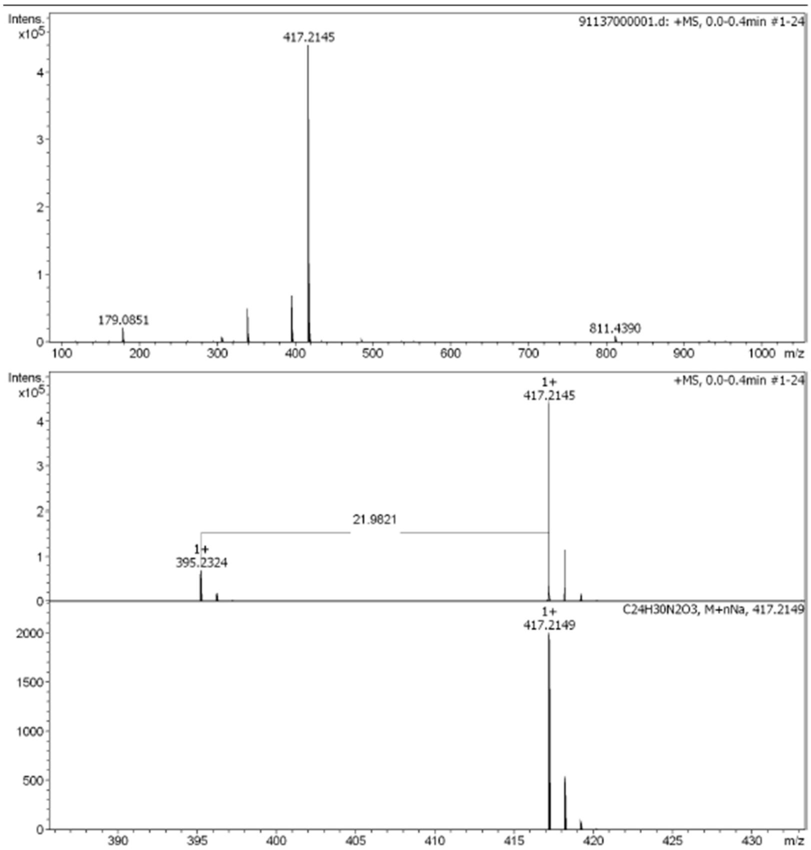

*Figure S4: HRMS spectrum of hydroxylimine **3**$_{IM}$. HRESI-MS m/z found (calculated) for $C_{24}H_{30}N_2O_3$: $[M+Na]^+$ 417.2145 (417.2149). Delta deviation: -0.96 ppm.*

2.2 **Hydroxylamine 3**

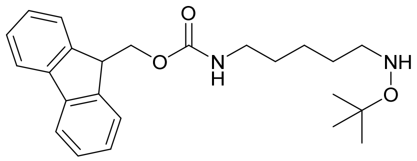

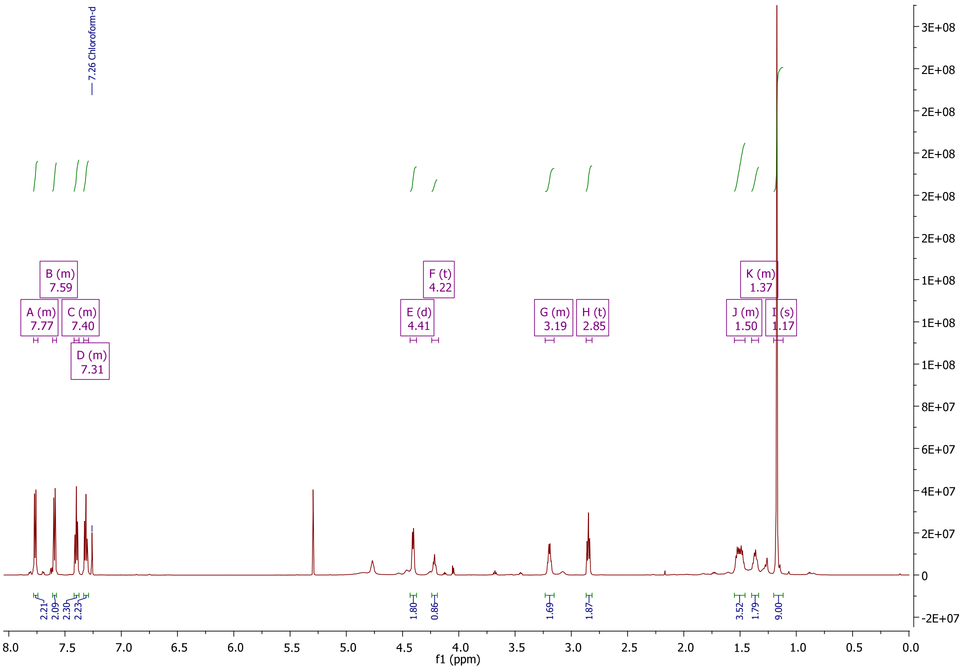

*Figure S5: ^{1}H-NMR (600.25 MHz, CDCl$_3$) spectrum of hydroxylamine **3.***

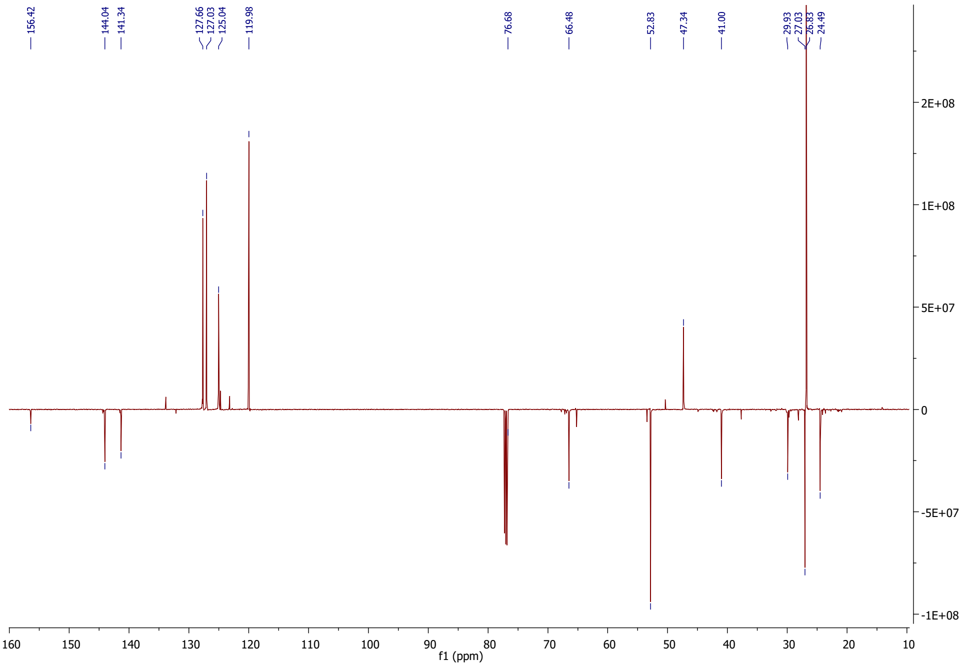

*Figure S6: ^{13}C-NMR (151 MHz, CDCl$_3$, DEPTq135) spectrum of hydroxylamine **3.***

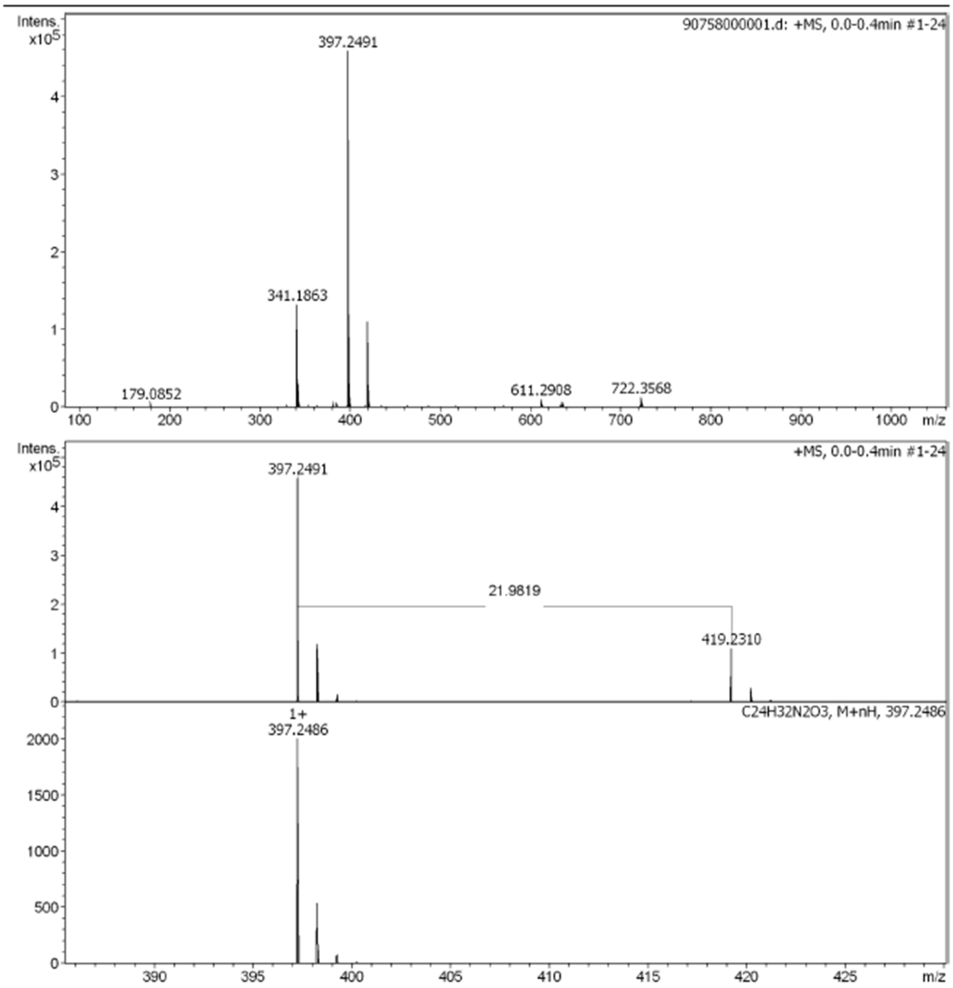

Figure S7: HRMS spectrum of hydroxylamine ***3.*** *HRESI-MS m/z found (calculated) for* $C_{24}H_{32}N_2O_3$*: $[M+H]^+$ 397.2491 (397.2486). Delta deviation: +1.26 ppm.*

2.3 **Fmoc-mon(*t*Bu) 4**

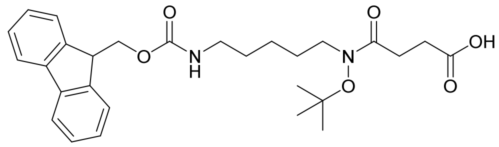

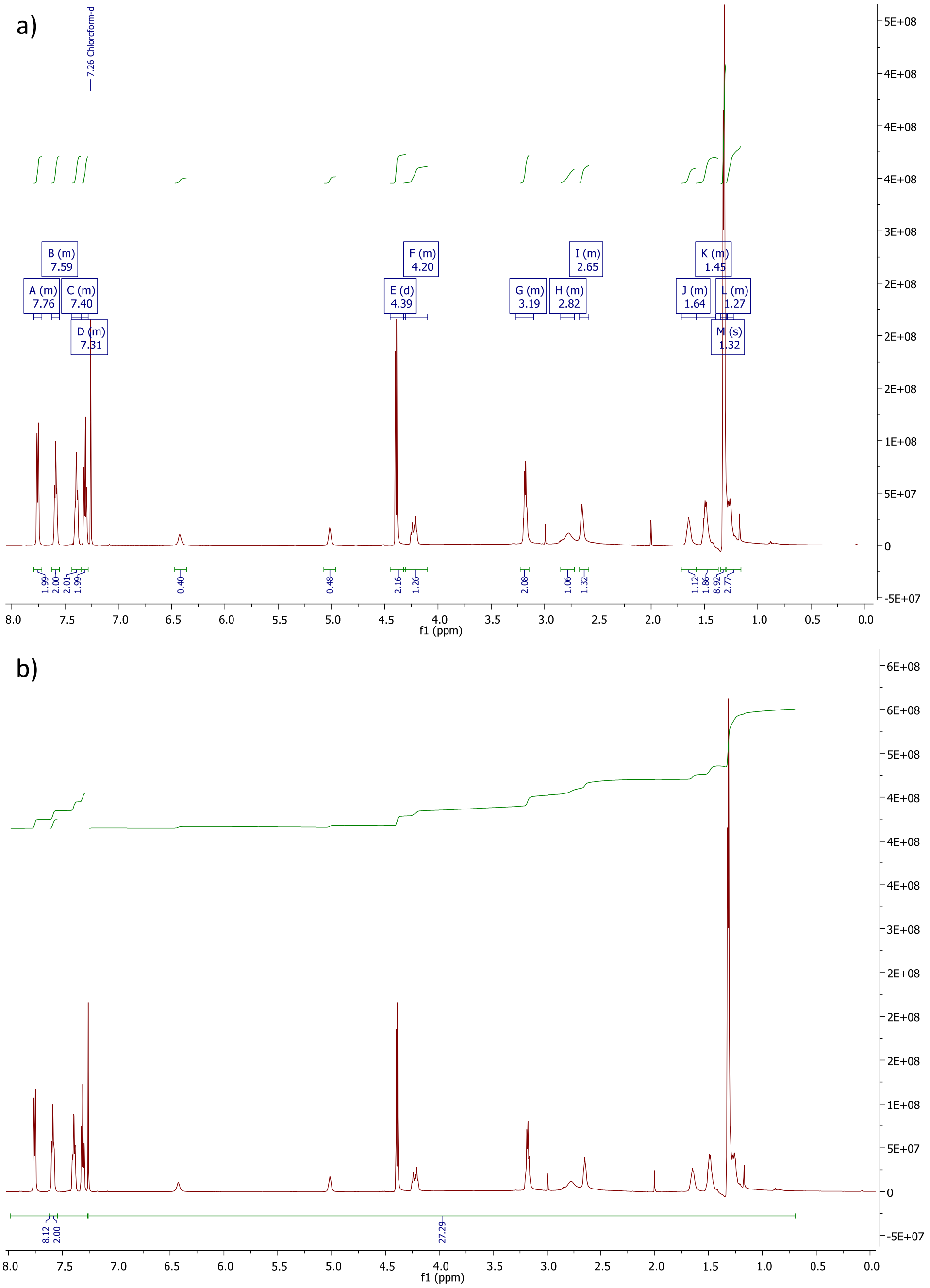

*Figure S8: ^{1}H-NMR (600.18 MHz, $CDCl_3$) spectrum of Fmoc-mon(tBu) **4**. a) Integration of all signals. Due to overlapping signals not all CH_2 groups could be observed. b) Integration of the complete spectrum, excluding the solvent signal. The aromatic signal was used for calibration (2.0 protons). The integration of the complete spectrum confirms the presence of all protons.*

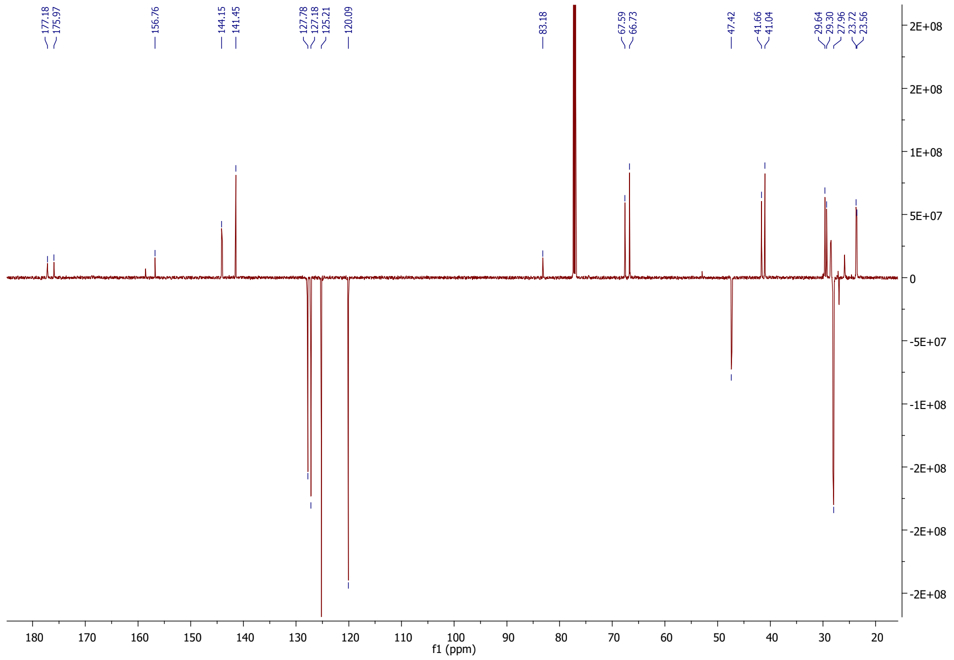

Figure S9: ^{13}C-NMR (151 MHz, $CDCl_3$, DEPTq135) spectrum of Fmoc-mon(tBu) ***4.***

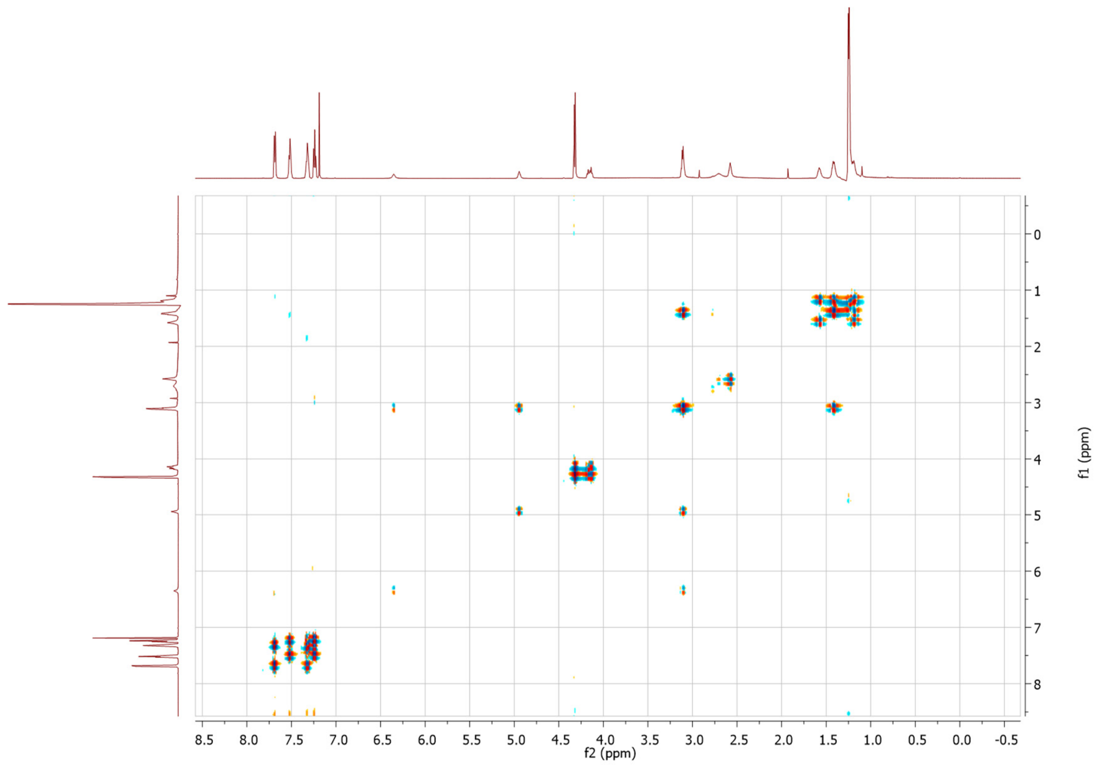

Figure S10: COSY-NMR ($CDCl_3$) spectrum of Fmoc-mon(tBu) ***4.***

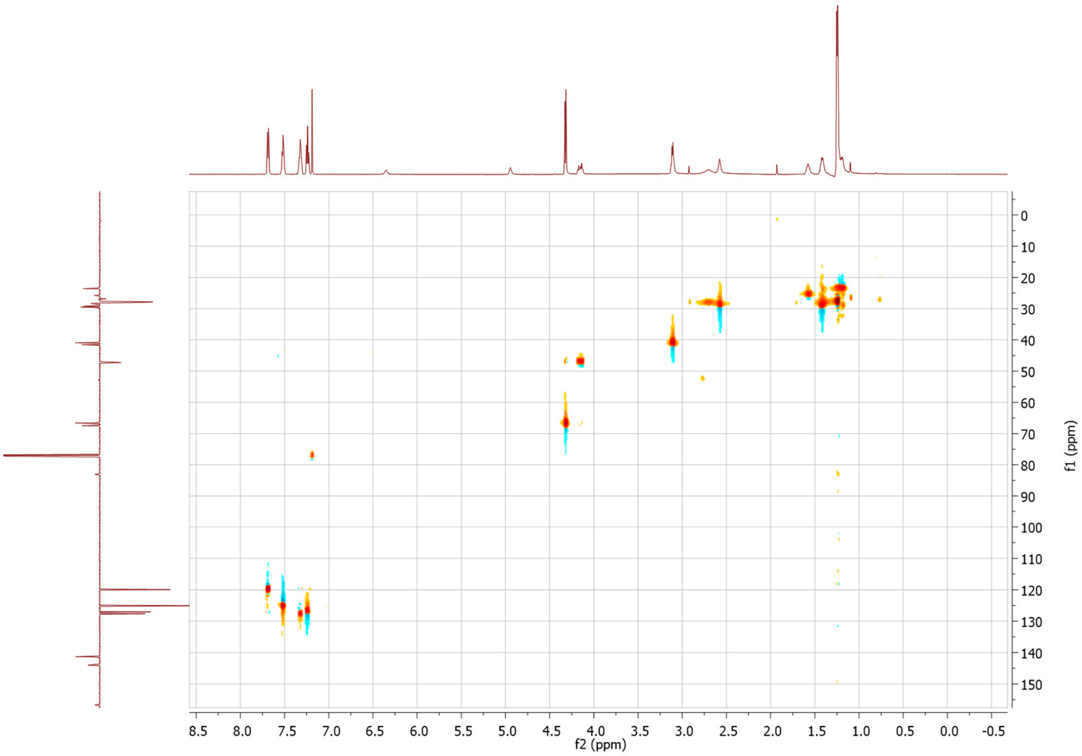

Figure S11: HSQC-NMR ($CDCl_3$) spectrum of Fmoc-mon(tBu) ***4.***

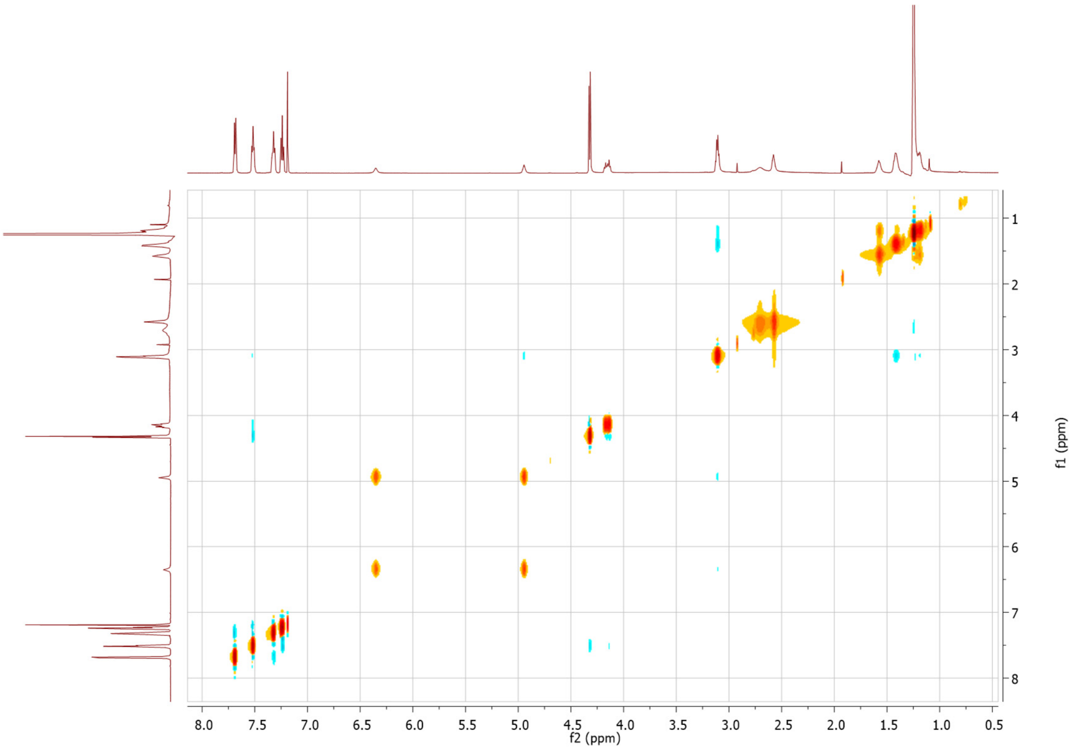

Figure S12: NOESY-NMR ($CDCl_3$) spectrum of Fmoc-mon(tBu) ***4.***

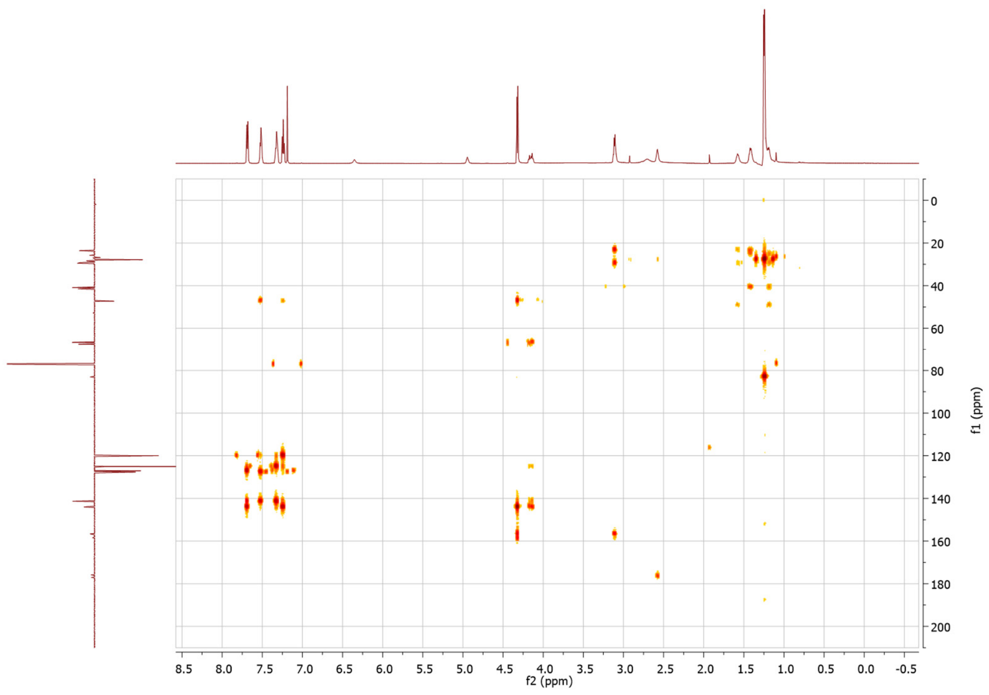

Figure S13: HMBC-NMR ($CDCl_3$) spectrum of Fmoc-mon(tBu) ***4.***

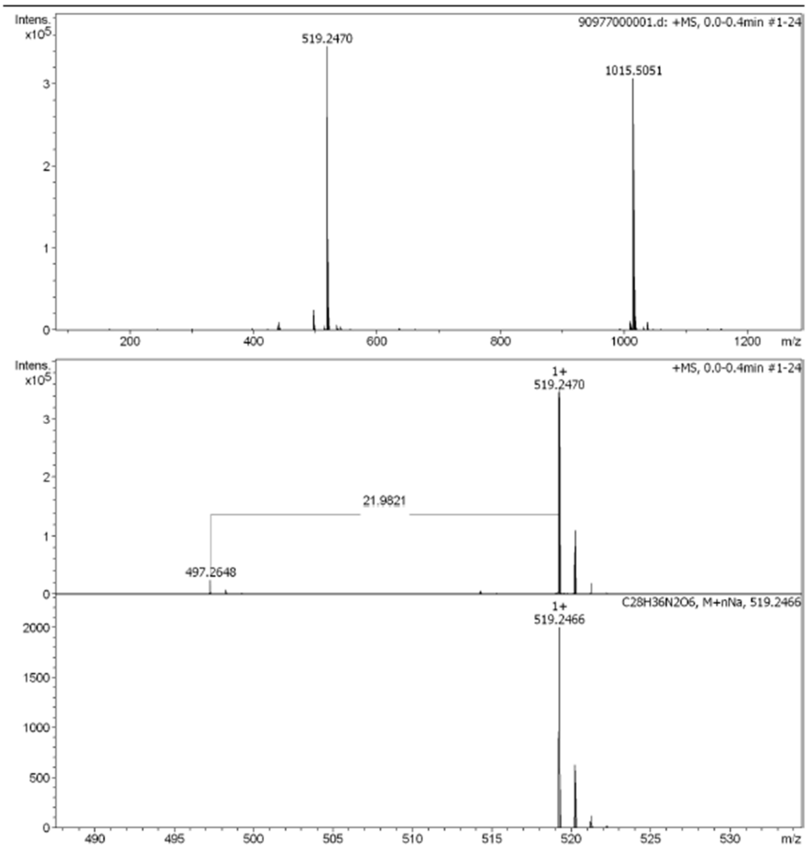

Figure S14: HRMS spectrum of Fmoc-mon(tBu) ***4.*** *HRESI-MS m/z found (calculated) for $C_{28}H_{36}N_2O_6$: $[M+Na]^+$ 519.2470 (519.2466). Delta deviation: +0.77 ppm.*

Part 3: Synthesis and analysis of the chelator DFO*[12] **5**

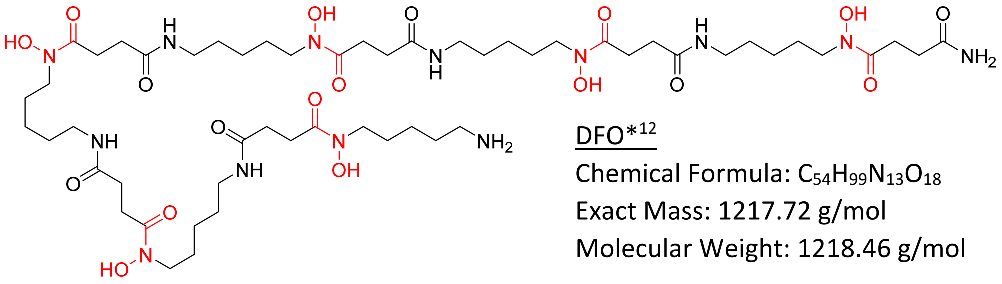

DFO*[12]
Chemical Formula: $C_{54}H_{99}N_{13}O_{18}$
Exact Mass: 1217.72 g/mol
Molecular Weight: 1218.46 g/mol

Table S1: Tested coupling reaction conditions. The yield was determined using the UV-VIS measurement of the cleaved Fmoc, Dibenzofulvene. Eq = equivalents, RT = Room Temperature.

	Eq	*Eq*	*Eq*	*Eq*	*Eq*	*Eq*
Resin (rink amid)	1,0	1	1	1	1	1
Monomer	1,0	3	5	5	5	5
HATU	0,9	5	5	5	5	5
DIPEA	0,36	5	8	8	8	8
Lutidine	0,54	-	-	-	-	-
**Scale**	0,05 mmol	0,02 mmol				
**Temperatur**	RT	50 °C	50 °C	50 °C	60 °C	65 °C
**Time**	60 min	40 min	40 min	60 min	45 min	60 min
**Yield**	8%	6%	38%	50%	55%	46%

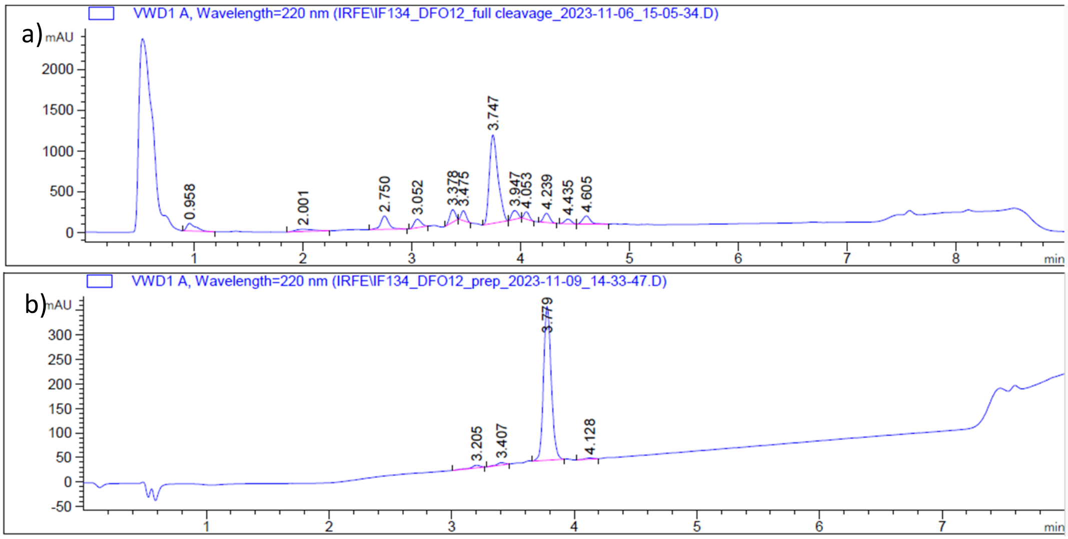

Figure S15: LC chromatogram of a) crude DFO[12] **5** after cleavage and b) purified DFO*[12] **5** via preparative HPLC. Note the different time scale of the 2 chromatograms.*

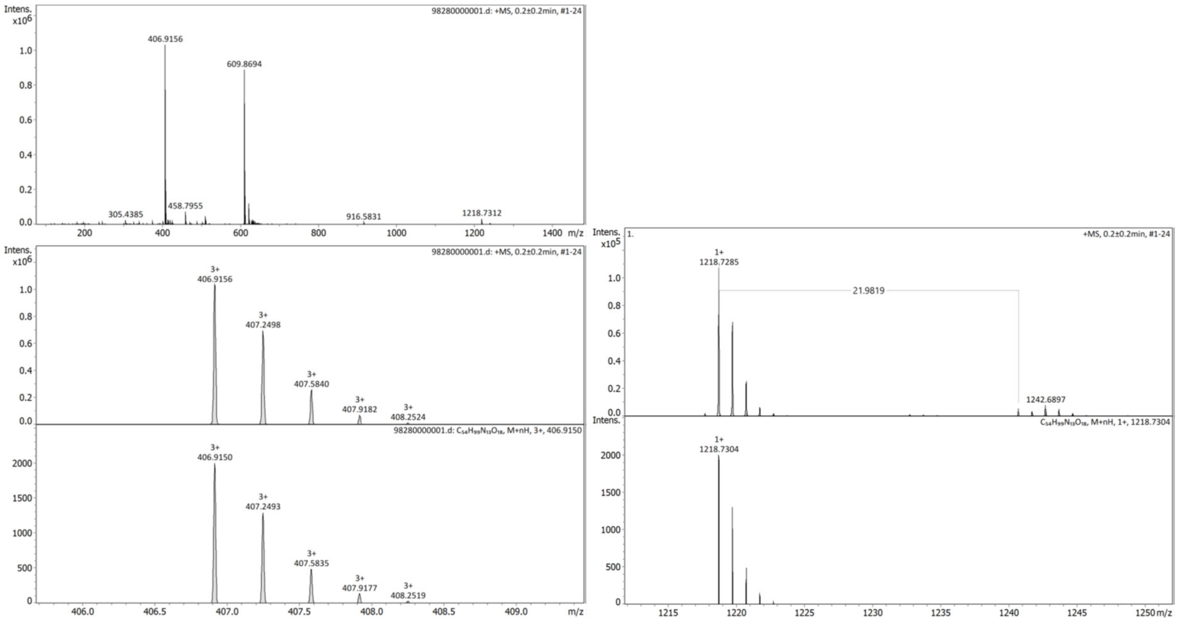

Figure S16: HRMS spectrum of DFO[12] **5**. HRESI-MS m/z found (calculated) for $C_{54}H_{99}N_{13}O_{18}$: $[M+H]^+$ 1218.7285 (1218.7304). Delta deviation: -1.56 ppm.*

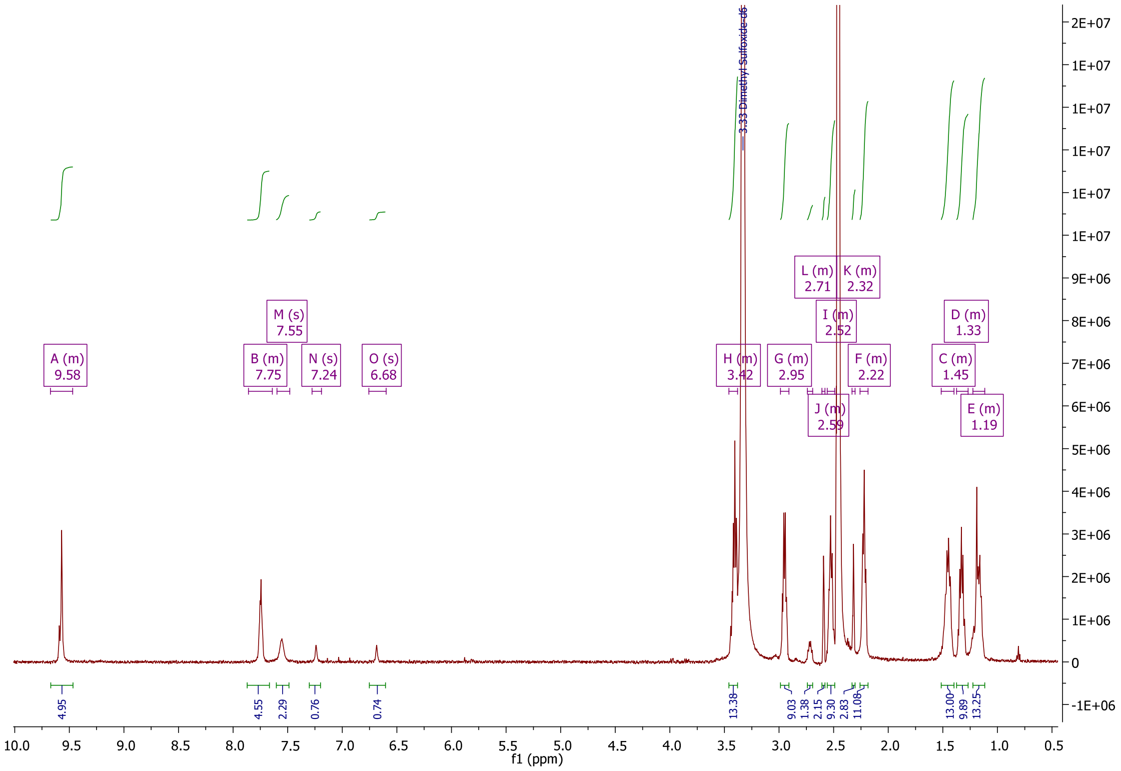

Figure S17: ^{1}H-NMR (500.1 MHz, DMSO) spectrum of DFO[12] **5**.*

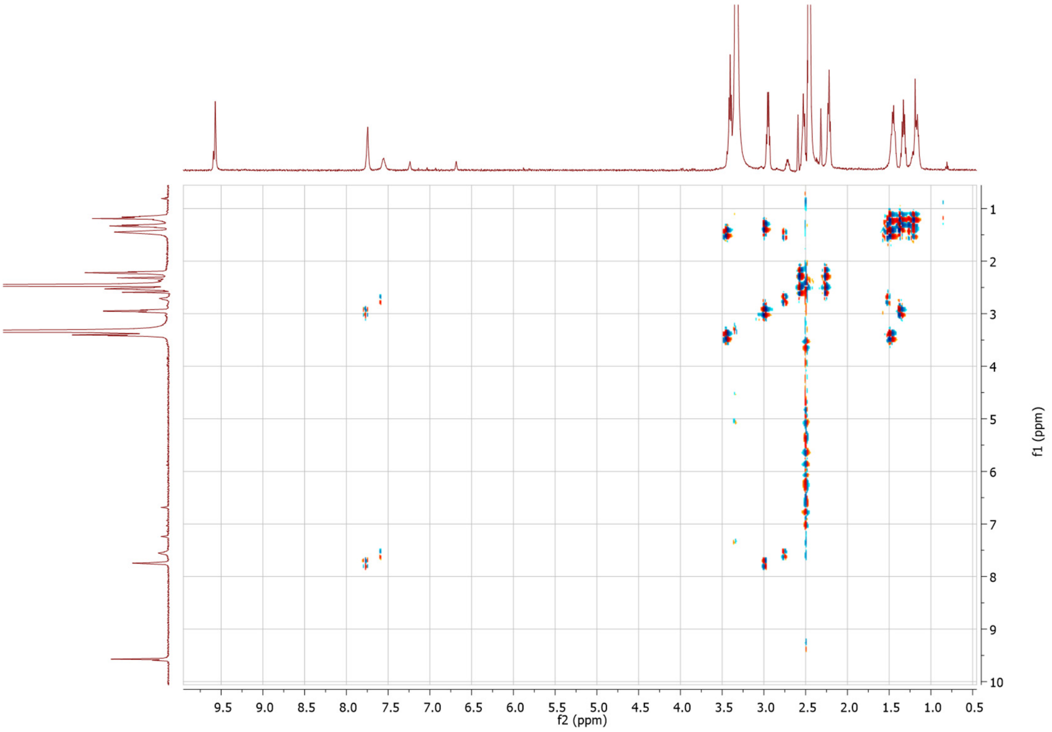

Figure S18: COSY-NMR (CDCl3) spectrum of DFO[12] **5**.*

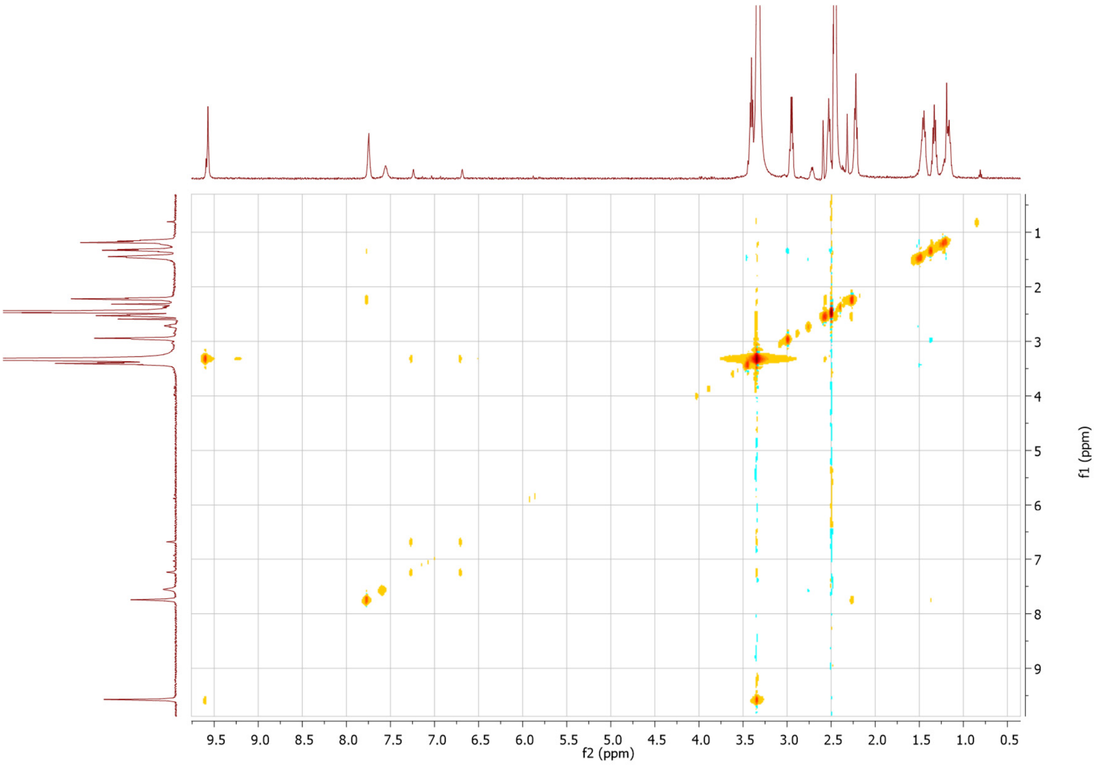

Figure S19: NOESY-NMR (CDCl3) spectrum of DFO[12] ***5***.

Part 4: RadioTLC chromatogram of [^{225}Ac]Ac-DFO*[12]

4.1 Radiolabeling

Table S2: Conditions and results of radiolabeling reactions.

Buffer	*pH*	*Temperature (°C)*	*Time (h)*	*RCY*
NaOAc (1.5 M)	4,5	60	3.5	quant.
NaOAc (1.5 M)	4,5	60	0.5	quant.
NaOAc (1.5 M)	4,5	37	0.5	quant.
NH_4OAc (1 M)	7,3	60	3.5	-
NH_4OAc (1 M)	7,3	60	24.0	-
HEPES (0.5 M)	7,4	60	3.5	-
HEPES (0.5 M)	7,4	60	24.0	-
TRIS (1 M)	7,4	60	3.5	12%
TRIS (1M)	8,5	60	3.5	quant.
TRIS (1M)	8,5	60	0.5	quant.
TRIS (1M)	8,5	37	0.5	quant.

All radio-TLC chromatograms with iTLC-SG in 50 mM EDTA. Signal at $R_f = 0$ represents ^{225}Ac-chelator. Signal at $R_f = 1$ represents [^{225}Ac]Ac-EDTA.

[^{225}Ac]Ac-DFO*[12]

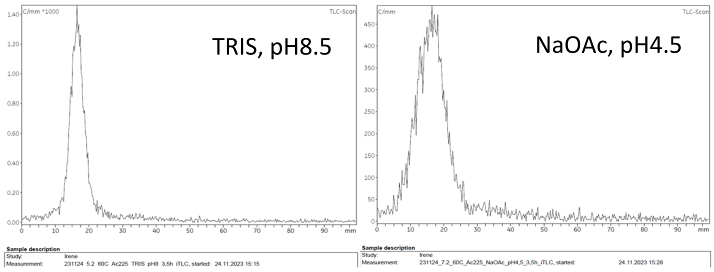

*Figure S20: Labelling solutions of **[^{225}Ac]Ac-DFO*[12]** in TRIS-buffer (1 M, pH 8.5, left) or NaOAc-buffer (1.5 M, pH 4.5, right) after 30 min at 37 °C.*

Negative control – free [^{225}Ac]$AcCl_3$

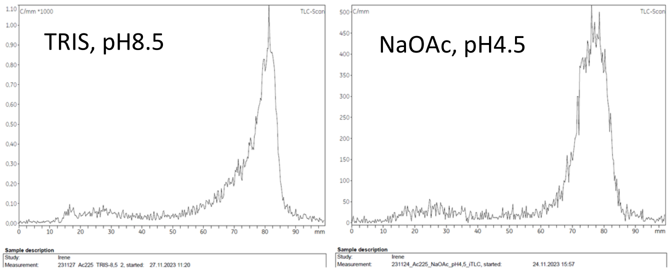

*Figure S21: **[^{225}Ac]$AcCl_3$** in TRIS-buffer (1 M, pH 8.5, left) or NaOAc-buffer (1.5 M, pH 4.5, right). Signal at $R_f = 1$ represents [^{225}Ac]Ac-EDTA.*

Positive control – [^{225}Ac]Ac-MacroPa

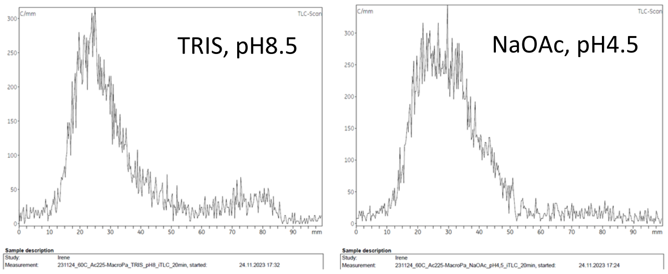

*Figure S22: Labelling solutions of **[^{225}Ac]Ac-MacroPa** in TRIS-buffer (1 M, pH 8.5, left) or NaOAc-buffer (1.5 M, pH 4.5, right) after 20 min.*

4.2 Stability of **[^{225}Ac]Ac-DFO^{*12}**

All radio-TLC chromatograms with iTLC-SG in 50 mM EDTA. Signal at R_f = 0 represents ^{225}Ac-chelator. Signal at R_f = 1 represents [^{225}Ac]Ac-EDTA.

In PBS

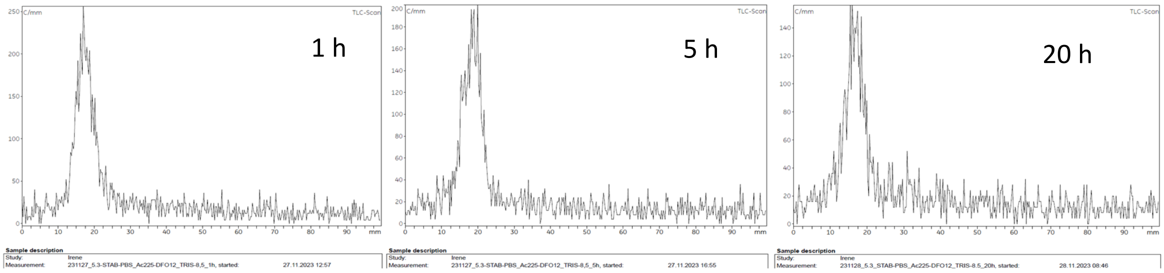

*Figure S23: Stability assay in PBS of **[^{225}Ac]Ac-DFO^{*12}** (radiolabeled in TRIS-buffer) at 37 °C at different time points.*

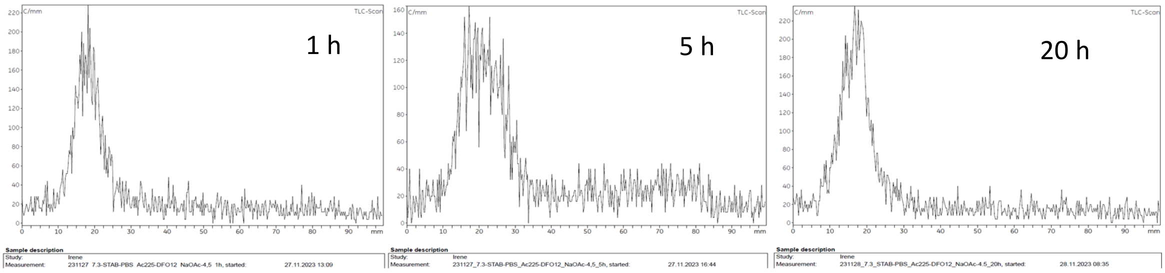

*Figure S24: Stability assay in PBS of **[^{225}Ac]Ac-DFO^{*12}** (radiolabeled in NaOAc-buffer) at 37 °C at different time points.*

In human serum

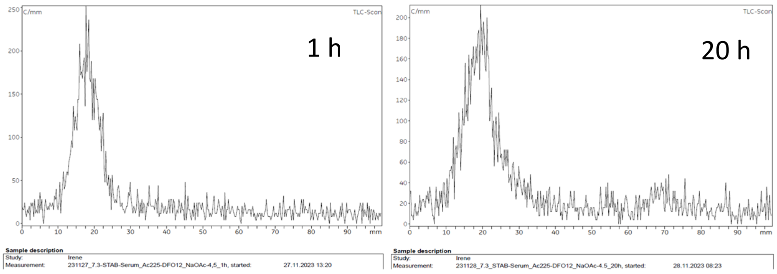

*Figure S25: Stability assay in human serum of **[^{225}Ac]Ac-DFO^{*12}** (radiolabeled in NaOAc-buffer) at 37 °C at different time points.*
